# Characterization of the antispike IgG immune response to COVID-19 vaccines in people with a wide variety of immunodeficiencies

**DOI:** 10.1126/sciadv.adh3150

**Published:** 2023-10-12

**Authors:** Mackenzie Zendt, Fausto A. Bustos Carrillo, Sophie Kelly, Taylor Saturday, Maureen DeGrange, Anita Ginigeme, Lurline Wu, Viviane Callier, Ana Ortega-Villa, Mondreakest Faust, Emma Chang-Rabley, Kara Bugal, Heather Kenney, Pavel Khil, Jung-Ho Youn, Gloria Osei, Pravesh Regmi, Victoria Anderson, Marita Bosticardo, Janine Daub, Thomas DiMaggio, Samantha Kreuzburg, Francesca Pala, Justina Pfister, Jennifer Treat, Jean Ulrick, Maria Karkanitsa, Heather Kalish, Douglas B. Kuhns, Debra L. Priel, Danielle L. Fink, John S. Tsang, Rachel Sparks, Gulbu Uzel, Meryl A. Waldman, Christa S. Zerbe, Ottavia M. Delmonte, Jenna R. E. Bergerson, Sanchita Das, Alexandra F. Freeman, Michail S. Lionakis, Kaitlyn Sadtler, Neeltje van Doremalen, Vincent Munster, Luigi D. Notarangelo, Steven M. Holland, Emily E. Ricotta

**Affiliations:** ^1^ Laboratory of Clinical Immunology and Microbiology, Division of Intramural Research (DIR), National Institute of Allergy and Infectious Diseases (NIAID), National Institutes of Health (NIH), Bethesda, MD, USA.; ^2^ Office of Data Science and Emerging Technologies, Office of Science Management and Operations, NIAID, NIH, Rockville, MD, USA.; ^3^ Trans-NIH Shared Resource on Biomedical Engineering and Physical Science, National Institute of Biomedical Imaging and Bioengineering (NIBIB), NIH, Bethesda, MD, USA.; ^4^ Laboratory of Virology, DIR, NIAID, NIH, Hamilton, NY, USA.; ^5^ Leidos Biomedical Research Inc., Frederick National Laboratory for Cancer Research, Frederick, MD, USA.; ^6^ Medical Science and Computing LLC, Rockville, MD, USA.; ^7^ Clinical Monitoring Research Program Directorate, Frederick National Laboratory for Cancer Research, Frederick, MD, USA.; ^8^ Biostatistics Research Branch, Division of Clinical Research, NIAID, NIH, Rockville, MD, USA.; ^9^ Section for Immunoengineering, NIBIB, NIH, Bethesda, MD, USA.; ^10^ Division of Laboratory Medicine, NIH Clinical Center, Bethesda, MD,USA.; ^11^ Department of Immunobiology and Yale Center for Systems and Engineering Immunology, Yale School of Medicine, New Haven, CT, USA.; ^12^ Department of Biomedical Engineering, Yale University, New Haven, CT,USA.; ^13^ Laboratory of Immune System Biology, DIR, NIAID, NIH, Bethesda, MD,USA.; ^14^ Kidney Disease Section, Kidney Diseases Branch, National Institute of Diabetes and Digestive and Kidney Diseases, NIH, Bethesda, MD, USA.

## Abstract

Research on coronavirus disease 2019 vaccination in immune-deficient/disordered people (IDP) has focused on cancer and organ transplantation populations. In a prospective cohort of 195 IDP and 35 healthy volunteers (HV), antispike immunoglobulin G (IgG) was detected in 88% of IDP after dose 2, increasing to 93% by 6 months after dose 3. Despite high seroconversion, median IgG levels for IDP never surpassed one-third that of HV. IgG binding to Omicron BA.1 was lowest among variants. Angiotensin-converting enzyme 2 pseudo-neutralization only modestly correlated with antispike IgG concentration. IgG levels were not significantly altered by receipt of different messenger RNA–based vaccines, immunomodulating treatments, and prior severe acute respiratory syndrome coronavirus 2 infections. While our data show that three doses of coronavirus disease 2019 vaccinations induce antispike IgG in most IDP, additional doses are needed to increase protection. Because of the notably reduced IgG response to Omicron BA.1, the efficacy of additional vaccinations, including bivalent vaccines, should be studied in this population.

## Teaser

After three COVID-19 vaccinations, immune-deficient participants had ≤33% of the anti-spike IgG antibody levels in healthy people.

## INTRODUCTION

The coronavirus disease 2019 (COVID-19) pandemic has heavily affected immune-deficient/disordered people (IDP), many of whom have a higher risk of experiencing worse COVID-19–related outcomes than the general population (*1*–*5*). COVID-19 vaccines significantly lower the risk of infection, transmission, severe disease, and death (*6*). However, the vulnerable IDP population, including those taking immunosuppressants, was excluded from all initial COVID-19 vaccine trials.

Most research on COVID-19 vaccination among IDP focuses on seroconversion rates after the first two doses, overlooks inborn errors of immunity (IEIs), and assesses populations with homogeneous immunodeficiencies, thus limiting its generalizability (*7*–*11*). Longitudinal studies evaluating antibody durability and characterizing the full effects of COVID-19 vaccination through 6 months after the third dose have been limited in IDP. While studies in the general population have measured waning vaccine-induced humoral immunity (*12*), severe acute respiratory syndrome coronavirus 2 (SARS-CoV-2) variant neutralization (*13*, *14*), and breakthrough infection rates (*15*), few studies quantify these outcomes in IDP.

Here, we characterize the immune response to COVID-19 vaccines in a large longitudinal cohort of IDP with a wide spectrum of immune disorders and healthy volunteers (HVs; the control group) by measuring postvaccination antibody concentrations, percent angiotensin-converting enzyme 2 (ACE2) inhibition (pseudo-neutralization capacity) against variants of concern, breakthrough infections, and vaccine-related adverse events from prevaccination up to 6 months after dose 3.

## RESULTS

### Participant characteristics

During the study period of 29 April 2021 to 28 April 2022, we followed 195 IDP and 35 HVs in the United States. Participants were 43.4 years old (range, 6 to 89) on average, and 146 (63.5%) were female. IDP were classified into five subgroups: antibody deficiencies (*n* = 52; e.g., common variable immunodeficiency (CVID) and hypogammaglobulinemia), primary immune regulation disorder (PIRD) (*n* = 46; e.g., autoimmune polyendocrinopathy candidiasis ectodermal dystrophy (APECED) and signal transducer and activator of transcription 3 dominant-negative disease), combined immunodeficiencies (*n* = 9; e.g., DOCK8 deficiency and severe combined immune deficiency), other IEIs (*n* = 26; e.g., chronic granulomatous disease, deficiency of adenosine deaminase 2 (DADA2) deficiency, and Good syndrome), and other immune disorders (*n* = 62; e.g., autoimmune disorders and solid organ transplant recipients) (Table 1 and table S1). Among IDP, 65 (33.3%) reported receipt of immunoglobulin replacement therapy (IgRT), 49 (25.1%) reported use of nonsteroidal immunosuppressive medications, and 63 (32.3%) received corticosteroids.

**Table 1.** Participant characteristics.

	Overall *N* (%)	HVs *N* (%)	IDPs *N* (%)
*N*	230	35	195
Age [mean (SD)]	43.4 (18.6)	39.7 (18.8)	44.1 (18.5)
Female sex	146 (63.5)	24 (68.6)	122 (62.6)
Immunological subgroup			
HV	35 (15.2)	35 (100.0)	0 (0.0)
Antibody deficiency	52 (22.6)	0 (0.0)	52 (26.7)
PIRD	46 (20.0)	0 (0.0)	46 (23.6)
Combined immunodeficiency	9 (3.9)	0 (0.0)	9 (4.6)
Other IEIs	26 (11.3)	0 (0.0)	26 (13.3)
Other immune disorders	62 (27.0)	0 (0.0)	62 (31.8)
Race			
Asian/Native Hawaiian/Pacific Islander	12 (5.2)	2 (5.7)	10 (5.1)
Black	10 (4.3)	4 (11.4)	6 (3.1)
Multiple Race	6 (2.6)	3 (8.6)	3 (1.5)
Unknown	7 (3.0)	1 (2.9)	6 (3.1)
White	195 (84.8)	25 (71.4)	170 (87.2)
Ethnicity			
Hispanic or Latino	15 (6.5)	2 (5.7)	13 (6.7)
Neither Hispanic nor Latino	204 (88.7)	30 (85.7)	174 (89.2)
Unknown	11 (4.8)	3 (8.6)	8 (4.1)
Medical status			
Other immunological condition	48 (23.1)	3 (8.6)	45 (23.1)
Seasonal allergies	105 (45.7)	22 (62.9)	83 (42.6)
Chronic lung disease	44 (21.2)	3 (8.6)	41 (21.0)
Diabetes mellitus	13 (5.7)	1 (2.9)	12 (6.2)
Cardiovascular disease	10 (4.8)	0 (0.0)	10 (5.1)
Kidney disease	24 (11.5)	0 (0.0)	24 (12.3)
Neurological developmental disorder	24 (10.4)	0 (0.0)	24 (12.3)
Other chronic disease	92 (40.0)	5 (14.3)	87 (44.6)
Immunomodulating medications			
IgRT	65 (28.3)	0 (0.0)	65 (33.3)
Nonsteroidal immunosuppressants	49 (21.3)	0 (0.0)	49 (25.1)
Corticosteroids	66 (28.7)	3 (8.6)	63 (32.3)
Brand of primary series			
JNJ-78436735 (Janssen)	4 (1.7)	2 (5.7)	2 (1.0)
mRNA-1273 (Moderna)	83 (36.1)	13 (37.1)	70 (35.9)
BNT162b2 (Pfizer-BioNTech)	143 (62.2)	20 (57.1)	123 (63.1)
Brand of third dose			
JNJ-78436735 (Janssen)	5 (2.2)	0 (0.0)	5 (2.6)
mRNA-1273 (Moderna)	71 (30.7)	13 (37.1)	58 (29.7)
BNT162b2 (Pfizer-BioNTech)	100 (43.5)	7 (20.0)	93 (47.7)
None	54 (23.5)	15 (42.9)	39 (20.0)
Same vaccine brand for doses 1 to 3	195 (84.8)	31 (88.6)	164 (84.1)

We collected prevaccination (baseline) blood samples as well as 1- and 6-month postvaccination samples through 6 months after dose 3 (fig. S1). Because of participants’ heterogeneous vaccine status upon enrollment, not all requested samples were available (table S2). By the end of the study period, 228 (99.1%) participants had received at least two vaccine doses, and 185 (80.4%) had received three doses. BNT162b2 (Pfizer-BioNTech) was received by 143 (62.2%) participants, 83 (36.1%) received mRNA-1273 (Moderna), and four (1.7%) received JNJ-78436735 (Janssen) for their primary series. Thirty-five (15.2%) participants switched vaccine brand during the study period (fig. S2 and table S3). The median time between the second and third doses was 175 days for IDP and 227 for HVs (*P* < 0.001) (fig. S3).

### Quantifying immunoglobulin G concentrations

**Fig. 1.**
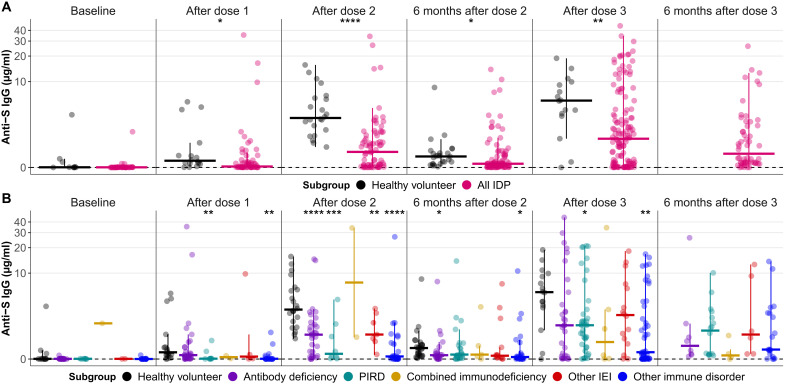
Anti-S IgG antibody titers before and after vaccination in HVs and IDP. Vertical bars extend from the median (horizontal bars) to the end of a standard boxplot’s whiskers. Within each time point, IDP subgroups are compared to HVs with Wilcoxon rank sum tests. By convention, *P ≤ 0.05, **P ≤ 0.01, ***P ≤ 0.001, and ****P ≤ 0.0001. No HV samples were available at 6 months after dose 3 as they were not eligible to receive a third dose until late 2021, after the study period. The assay limit of detection is shown with a dashed line. Additional data (relative and raw numbers) corresponding to this figure are presented in tables S4 and S5. (**A**) Anti-S IgG titers for HVs (black, *n* = 35) and all IDP (pink, *n* = 195). *P* values obtained from Wilcoxon rank sum tests. (**B**) Anti-S IgG for HVs (black, *n* = 35) and IDP subgroups: antibody deficiencies (purple, *n* = 52), PIRD (teal, *n* = 46), combined immunodeficiency (yellow, *n* = 9), other IEIs (red, *n* = 26), and other immune disorders (blue, *n* = 62).

Antispike (anti-S) immunoglobulin G (IgG) was detected in 87.5% of IDP 1 month after dose 2 by enzyme-linked immunosorbent assay (ELISA), with increasing seroconversion rates at all subsequent time points (92.0% 6 months after dose 2, 92.4% 1 month after dose 3, and 92.6% 6 months after dose 3). Anti-S IgG was detected in 100% of HVs 1 and 6 months after dose 2 and 93.8% of HVs after dose 3. Anti-S IgG concentrations in IDP subgroups followed the same trajectory as HVs: rising after dose 2, declining by 77% 6 months later, and rebounding after a third dose beyond post–dose 2 levels (Fig. 1A). In a paired analysis among IDP, evidence of a significant difference in anti-S IgG concentrations 1 month after dose 3 and 6 months later was lacking (*P* = 0.599).

Despite comparable antibody dynamics, IDP exhibited significantly lower levels of anti-S IgG than HVs at each postbaseline time point where a comparison was possible (Fig. 1A). Across time points, the “Other immune disorders” subgroup tended to have the lowest median IgG levels relative to HVs; even after three doses, they only exhibited 6.3% of median HV concentrations (Fig. 1B and tables S4 and S5). After dose 3, median IgG concentrations across IDP subgroups were ≤50% of median HV concentrations. Collectively, median IDP concentrations were always ≤33% of median HV concentrations (table S4).

### Variant binding and ACE2 pseudo-neutralizing capacity (% inhibition)

**Fig. 2.**
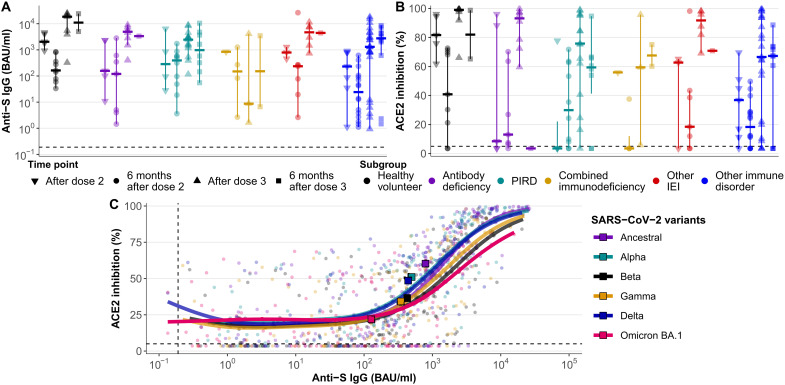
Anti-S IgG antibody concentration and ACE2 pseudo-neutralization. Anti-S IgG antibody concentration and ACE2 pseudo-neutralization capacity (% inhibition) for participants (*n* = 105) as measured by ECL. Samples corresponding to participants with evidence of prior SARS-CoV-2 infection and those on AZD7442 (tixagevimab-cilgavimab, AstraZeneca), and postbreakthrough infection data were removed to quantify the impact of the vaccines against an immunologically naïve background. Dashed lines indicate assay limits of detection. Three outliers were removed from this chart for visualization purposes. (**A**) Anti-S IgG concentration against the ancestral strain for each subgroup across time points; within each subgroup, data are temporally ordered from left (after dose 2) to right (6 months after dose 3). Vertical bars extend from the median (horizontal bars) to the end of a standard boxplot’s whiskers. Comparable data for the Omicron BA.1 variant are shown in fig. S4A. (**B**) ACE2 inhibition data against the ancestral strain for each subgroup across time points; within each subgroup, data are temporally ordered from left (after dose 2) to right (6 months after dose 3). Vertical bars extend from the median (horizontal bars) to the end of a standard boxplot’s whiskers. Comparable data for the Omicron BA.1 variant are shown in fig. S4B. (**C**) Generalized additive mixed model (GAMM) trendlines of ACE2 inhibition as functions of anti-S IgG antibody concentration. The model is based on the post–dose 2, 6-month post–dose 2, post–dose 3, and 6-month post–dose 3 samples for all six immunological subgroups (i.e., HVs, antibody deficiencies, PIRD, combined immunodeficiencies, other IEI, and other immune disorders). Median values are shown as squares.

We further measured anti-S IgG concentrations and ACE2 pseudo-neutralization capacity (% ACE2 inhibition by anti-S IgG) for multiple SARS-CoV-2 variants of concern via electrochemiluminescence (ECL). For these analyses, we examined the impact of the vaccines against an immunologically naïve background by removing samples from participants with evidence of prior SARS-CoV-2 infection and those on AZD7442 (tixagevimab-cilgavimab, AstraZeneca). We observed consistently robust anti-S IgG levels and ACE2 inhibition over time to multiple SARS-CoV-2 variants, with three exceptions. First and concordant with the ELISA data at 6 months after dose 2, anti-S IgG concentrations and ACE2 inhibition declined substantially (65 and 45%, respectively) across immunological subgroups and SARS-CoV-2 variants [Fig. 2, A and B (ancestral), and fig. S4 (Omicron BA.1)]. Second, anti-S IgG concentrations were similar across time points for all variants except Omicron BA.1, which were significantly lower at each time point relative to the other variants (fig. S5). Third, ACE2 inhibition of Omicron BA.1 was significantly lower at most time points against most variants (fig. S6). Notably, ACE2 inhibition levels of Omicron BA.1 were usually similar to, although lower than, those for the Beta and Gamma variants.

The overall correlation between anti-S IgG concentration and ACE2 inhibition was modestly positive [0.53; 95% confidence interval (CI), 0.46 to 0.59] and increased logistically (Fig. 2C). IDP subgroups had correlation values approximating HVs (0.57; 95% CI, 0.44 to 0.73) (table S6). The correlation for samples taken 1 month after vaccination was significantly higher than for samples taken 6 months afterward (*P* < 0.0001) (table S7). Correlation coefficients for the ancestral, Alpha, and Delta variants were nearly identical (~0.60) and were a bit higher than the Beta and Gamma variants (~0.52). However, Omicron BA.1 had a significantly lower (*P* < 0.0001) correlation than all other variants (0.25; 95% CI, 0.14 to 0.34) (table S8), and it exhibited the lowest correlation within each immune subgroup (table S9).

In addition, across large ranges of anti-S IgG concentrations, we observed decreased ACE2 inhibition against the Beta, Gamma, and especially Omicron BA.1 variants relative to the ancestral, Alpha, and Delta variants. For example, whereas all other variants achieved ≥91% mean inhibition at concentrations of 15,000 BAU (binding antibody units)/ml, the anti–Omicron BA.1 IgG concentration peaked at 15,000 BAU/ml, resulting in a maximum mean inhibition of only 82% (fig. S7). Critically, the Omicron BA.1 variant exhibited a substantially more attenuated association between IgG concentration and ACE2 inhibition than other variants. Consequently, it had significantly lower median concentration and inhibition values across the 10 comparisons to every other variant (Fig. 2C and fig. S8).

Across time points, ACE2 inhibition was ≤75% for most samples after dose 2 and 6 months later (fig. S9). A significantly higher percentage of post–dose 3 samples achieved >75% inhibition compared to post–dose 2 samples (*P* < 0.0001). While the highest percentage (40%) of samples achieving >75% inhibition was observed at post–dose 3, only 8% did so 6 months later (*P* < 0.0001), even at high anti-S IgG concentrations. Few IDP achieved IgG concentrations approximating that of HVs, leading to reduced ACE2 inhibition across IgG concentrations (fig. S10).

### Factors potentially affecting anti-S IgG levels

We examined multiple factors potentially affecting the anti-S IgG response. We found only three instances where antibody concentration significantly differed by vaccine brand: among the other immune disorder subgroup at the post–dose 1 and post–dose 3 time points and among HVs at the post–dose 3 time points. In these instances, antibody concentrations from mRNA-1273 recipients were higher than BNT162b2 recipients (fig. S11). Switching vaccine brand at dose 3 had no impact on antibody concentration in most subgroups. Only those in the “other IEI” subgroup who switched vaccine brands between doses 2 and 3 had a significantly lower anti-S IgG concentration after dose 3 than dose 2 (*P* = 0.039) (fig. S12). Because few IDP received the mRNA-1273 half-dose booster, a reliable comparison between full and half dose was not possible. In addition, so few participants received the JNJ-78436735 vaccine that we were unable to assess whether elicited antibody concentrations differed between its recipients and those of mRNA-based vaccines.

**Fig. 3.**
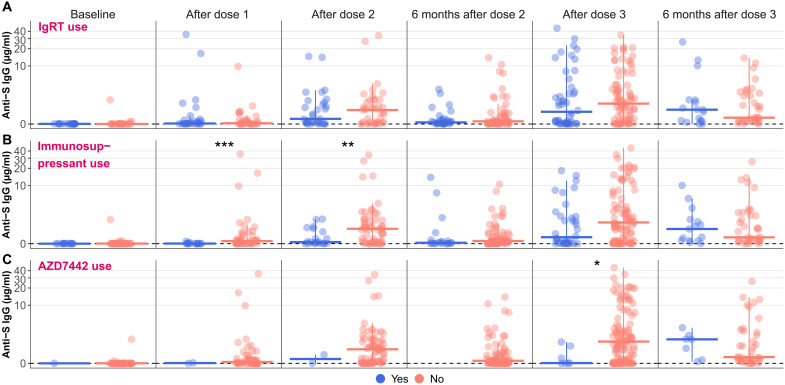
Anti-S IgG antibody concentration by immunomodulatory therapeutic use. Vertical bars extend from the median (horizontal bars) to the end of a standard boxplot’s whiskers. Data are shown for IDP (*n* = 195) across all time points. In all cases, missing values were coded as “No.” *P* values obtained from Wilcoxon rank sum tests. By convention, *P ≤ 0.05, **P ≤ 0.01, and ***P ≤ 0.001. The assay limit of detection is shown with a dashed line. (**A**) Participants were categorized by IgRT use at baseline. (**B**) Participants were categorized by immunosuppressant use at baseline. (**C**) Participants were categorized by ever use of AZD7442 (tixagevimab-cilgavimab, AstraZeneca) during the study period. Seven IDP received AZD7442, one participant between the 6-month post–dose 2 sample and the post–dose 3 sample, and six participants between the post–dose 3 sample and the 6-month post–dose 3 sample. No samples from participants who ever received AZD7442 were available at the 6-month post–dose 2 time point.

We found no significant difference in antibody concentrations at any time point among IDP receiving and not receiving IgRT (Fig. 3A). After excluding IgRT use, the other immune disorder subgroup still had the lowest concentration relative to HVs (fig. S13). Those on any type of immunosuppressant had significantly lower titers after dose 1 (*P* < 0.001) and dose 2 (*P* = 0.009), although there was no significant difference at other time points (Fig. 3B). Of the 15 participants who received rituximab (seven <6 months before dose 1, four within 6 to 12 months, and four >12 months), most had undetectable anti-S IgG, regardless of the time between their last rituximab infusion and first vaccine dose. Across time points, 11 of 15 had detectable anti-S IgG concentrations at least once. However, IgG levels were always low, usually falling below the positivity threshold (fig. S14). Seven IDP received AZD7442 during the study period, one before dose 3, and six between the post–dose 3 and 6-month post–dose 3 time points. All AZD7442 recipients had very low anti-S IgG at prereceipt time points, but after receipt, median anti-S IgG levels were similar to IDP not on AZD7442 (Fig. 3C).

The percentage of participants testing positive for SARS-CoV-2 antinucleocapsid (anti-N) IgG increased over the course of the study due to natural SARS-CoV-2 infection and IgRT use. Postvaccination anti-S IgG levels among those who were anti-N IgG-positive were significantly higher at baseline (*P* < 0.0001), post–dose 1 (*P* < 0.001), and 6-month post–dose 2 (*P* = 0.010) compared to anti-N IgG-negative participants (fig. S15). Between post–dose 3 and 6-month post–dose 3, when most breakthrough infections occurred, anti-S IgG concentrations between anti-N–positive and anti-N–negative participants were similar. Median anti-S IgG levels for IDP were alike after dose 3 and 6 months later, whether breakthrough infections were included (*P* = 0.599) or excluded (*P* = 0.547). Thus, we found that our main results were not significantly altered by participants’ use of different mRNA-based vaccines, immunomodulatory treatments/therapies, and prior SARS-CoV-2 infections.

### Breakthrough infections

**Fig. 4.**
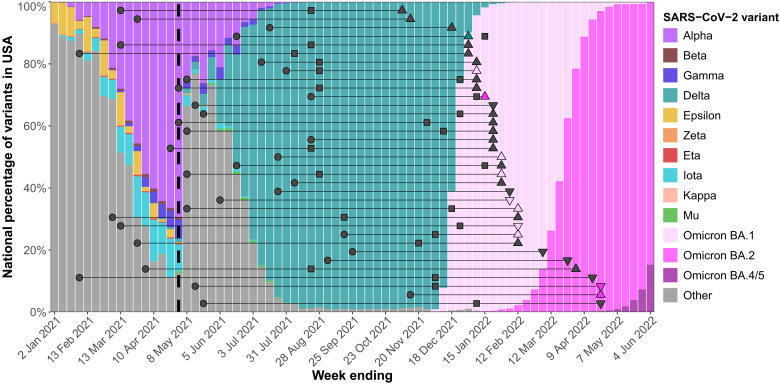
Swimmer plot of breakthrough infections relative to SARS-CoV-2 waves in the United States. The date of dose 2 receipt (circle), date of dose 3 receipt (square), and date of breakthrough infection detection (triangle) are shown for all 35 participants with breakthrough infections. Upright triangles represent IDP participants (*n* = 26), and upside-down triangles represent HV participants (*n* = 9). The color of the triangle corresponds to the known causative SARS-CoV-2 variant; infections for which the causative variant is unknown are shown in gray. The background colors show the national SARS-CoV-2 variant sampling percentages from the Centers for Disease Control and Prevention (CDC). The dashed line indicates the start of the study period, 29 April 2021. Tick marks on the *x* axis are placed at every fourth week (approximately a month’s worth of time).

Of 230 participants, 35 reported a positive SARS-CoV-2 test during the study period. Seventeen individuals were identified or confirmed by testing participant saliva that we received every 2 weeks, and the remainder was identified by external laboratories and/or home antigen tests. Breakthrough infections occurred in 26 (13.3%) IDP and 9 (25.7%) HVs (*P* = 0.105). All participants were infected between December 2021 and April 2022. Of the 35 breakthrough infections, 11 variants were identified by sequencing. Most infections were caused by the Omicron BA.1 variant (Fig. 4 and table S10). Median time between most recent vaccine dose and SARS-CoV-2 infection was 4.5 months for IDP and 5.4 months for HVs (*P* = 0.109). By the time that breakthrough infections occurred, 35 (100%) participants had received at least two vaccine doses, but only 23 (66%) had received a third. Only one participant, an IDP, was hospitalized for COVID-19. They were discharged without the need for intensive care or ventilation.

### Adverse events

IDP experienced similar postvaccination adverse events as HVs. Across 91 total vaccine doses in HVs and 608 doses in IDP, there were five postvaccine adverse events that required emergency room visits [four visits (0.6%) among three IDP and one visit (1.1%) in HVs; *P* = 0.432] (table S11 and fig. S16).

## DISCUSSION

This study demonstrates that COVID-19 vaccines are well tolerated and elicit anti-S IgG in all IDP groups, especially after three doses. A third dose substantially elevated antibody concentration in participants on immunosuppressants including in some participants receiving rituximab within 6 months of vaccination, which differs from other studies that documented low seroconversion in IDP on immunosuppressants after the initial two-dose primary series (*16*–*18*). Still, despite multiple doses of vaccination increasing antibody concentrations among IDP, their collective median concentration never exceeded 33% of the corresponding HV concentration at any time point. Combined with the discouragingly low ACE2 inhibition we observed against Omicron BA.1, our results reinforce the evidence that more than three doses are necessary for IDP to achieve similar levels of immunogenicity and SARS-CoV-2 inhibition as immunocompetent people with a comparable number of vaccine doses (*19*, *20*).

Use of the preexposure prophylactic AZD7442 (*21*) recapitulated anti-S IgG concentrations in nonresponders, emphasizing the utility of adjunctive prevention measures. Further study on the durability of this response is warranted, especially following the removal of its emergency use authorization due to decreased activity against newer SARS-CoV-2 variants (*22*). Participants receiving IgRT at any point before or during the study period likely acquired donor anti-S IgG, as anti-S IgG first appeared in the United States. IgRT pooled in September 2020 and rapidly increased afterward (*23*). Despite this, IgRT recipients did not display higher anti-S IgG antibody concentrations than non-IgRT recipients at any time point, supporting evidence that IgRT provides only limited passive immunity to immunologically vulnerable persons (*24*). Although self-reported treatment was a limitation in our study, use of IgRT and other immunomodulators did not appear to alter our conclusions.

Antibody concentrations in both IDP and HVs were high across all variants we measured except Omicron BA.1 for which a substantial drop was observed at all time points. This was predictable, as Omicron BA.1’s 37 spike mutations relative to the ancestral strain permit high ACE2 binding affinity while decreasing immune recognition (*25*–*27*). ACE2 inhibition against Omicron BA.1 failed to reach 100% even at high IgG antibody concentrations, a phenomenon observed across many Omicron subvariants (*28*), which, similar to the Beta and Gamma variants that also demonstrated lower inhibition (*29*, *30*), have been shown to escape from neutralizing antibodies elicited by the monovalent mRNA vaccines in both immunocompetent persons and IDP (*28*, *31*–*34*). The modestly positive correlation (*35*) between antibody concentration and ACE2 inhibition suggests that the quantity of anti-S IgG is only weakly indicative of inhibiting potential, particularly against Omicron BA.1. Given the increasing diversification of the Omicron lineage, we predict that the correlation of IgG concentration and ACE2 inhibition elicited by monovalent vaccines is even lower for post–Omicron BA.1 variants. Therefore, in the Omicron era, antibody concentration should not be used as a proxy for the level of neutralizing immunity. Our anti-S IgG concentration and ACE2 inhibition results are concordant with prior data showing that the ancestral strain is antigenically closer to Alpha; while Delta, Gamma, and Beta are similar to one another; and Omicron BA.1 is far from other variants in antigenic space (*36*, *37*). We were unable to examine the impact of the bivalent mRNA in this study as it was not available to the public during the study period. However, future reports from this cohort will examine anti-S IgG inhibition and the T cell response induced by the bivalent mRNA vaccines to many Omicron subvariants (*38*, *39*).

Despite lower vaccine effectiveness in IDP compared to the general population (*40*), fewer IDP than HVs experienced breakthrough infections in our study. This discrepancy may have resulted from preventative measures taken by IDP, including receipt of AZD7442 and ongoing behavioral changes such as mask wearing and social distancing (*41*–*43*). Most IDP received a full third dose in late 2021, shortly before the explosive Omicron BA.1 epidemic in the United States (*44*). Nevertheless, most breakthrough infections in this cohort were caused by Omicron subvariants. This finding highlights the reduced effectiveness of the original, mRNA-based vaccines against the Omicron lineage and emphasizes the need for updated multivalent vaccines. Our results suggest that Omicron BA.1’s higher capacity for immune evasion places IDPs who have only received the monovalent mRNA vaccines at higher risk for future SARS-CoV-2 infections with new subvariants, such as XBB, that have a high immune-evasion potential. A detailed investigation of breakthrough infections, their clinical severity, and postvaccination behavioral changes in this cohort will be assessed in future studies.

Overall, we demonstrate that across a wide variety of immune disorders, multiple doses of COVID-19 vaccines are safe and effective in terms of antibody concentration, ACE2 inhibition, and preventing breakthrough infections. Vaccine recommendations should be strengthened to reflect the strong evidence regarding the impact of COVID-19 vaccinations among IDP. Our results provide robust evidence that IDP should remain current on their COVID-19 vaccinations and require more doses than HVs to increase their anti-S IgG response. The introduction of the bivalent mRNA-based vaccines likely alters the vaccine-derived immunological landscape for vaccinees, including IDP. Future work, including from our cohort, will characterize its contours and the degree of protection afforded to this vulnerable population.

## MATERIALS AND METHODS

### Study design

We established a prospective cohort study of IDP and HVs from across the United States. Enrollment began in April 2021 after COVID-19 vaccines was widely available. All IDP had evidence of a primary or secondary immune disorder. HVs had no known immune disorder and included unaffected relatives of IDP who participated in the study. Participants were enrolled at various points in their vaccination schedule and received the U.S. Food and Drug Administration–authorized/approved vaccines externally to this study. While HVs were enrolled throughout the study period, blood samples could not be obtained for them at the 6- month post–dose 3 time point because HVs were not eligible to receive a third dose as soon as IDP, per contemporary vaccination guidelines from the Centers for Disease Control and Prevention (CDC). Thus, by the end of the study period, 6 months had not passed since the third dose for any HV. Pre–third dose samples were collected if the third dose occurred less than 6 months after the second dose; these samples (*n* = 33) were grouped with the 6-month post–dose 2 samples.

This study (NCT04852276) was approved by the Institutional Review Board of the National Institutes of Health (NIH) and was conducted in accordance with the Declaration of Helsinki and Good Clinical Practice guidelines. Adult participants and parents or legal guardians of pediatric participants provided written informed consent. Participants 6 years and older gave verbal assent.

### Blood collection methods

Blood was collected remotely via venous draw or by finger stick. Finger stick samples were collected as described previously (*45*, *46*) and directly stored at –80°C. Whole blood was collected from venous draw into EDTA and shipped overnight to the NIH for processing. Plasma was separated from peripheral blood samples (EDTA anticoagulant) by centrifugation for 10 min at 500*g*. The supernatant plasma was removed and spun a second time for 10 min to pellet residual platelets and leukocytes. The supernatant plasma was then transferred to 1.0-ml conical cryotubes and stored at –80°C. Baseline blood samples were collected in unvaccinated participants, when possible. Postvaccination samples were collected 1 and 6 months after vaccination.

### Enzyme-linked immunosorbent assays

ELISAs were performed as previously described (*45*–*47*) to quantify SARS-CoV-2 anti-S (*48*) and anti-N IgG antibody concentrations (*45*, *46*). Briefly, whole blood was eluted from one microsampler, and the subsequent eluate was used in a 96-well ELISA. Ancestral SARS-CoV-2 spike antigen [1 μg/ml; National Institute of Allergy and Infectious Diseases (NIAID) Vaccine Research Center] was diluted in 1× phosphate-buffered saline (PBS; Thermo Fisher Scientific) and added to each well of a 96-well plate (Nunc MaxiSorp) before incubation at 4°C overnight. All subsequent steps other than sample addition occurred on a BioTek EL406 with plate stacker to automate washing and blocking. Wells were washed with PBST (1× PBS + 0.05% Tween 20; three cycles) and then blocked for 2 hours in PBST + 5.0% nonfat dried milk. Wells were washed again, and eluate was diluted 1:10 in 1× PBS + 5.0% non-fat dry milk. Samples were then added to the plates and incubated for 1 hour. Wells were washed with PBST and then incubated with a 1:4000 dilution of horseradish peroxidase–conjugated goat anti-human IgG (H+L) secondary antibody in blocking buffer. Wells were washed then incubated with 1-Step Ultra TMB-ELISA Substrate Solution (Thermo Fisher Scientific) for 10 min before stopping the reaction with 1 N of sulfuric acid stop solution (Thermo Fisher Scientific). Plates were read within 30 min of stopping the reaction on a BioTek Epoch2 Plate reader at 450 and 650 nm. Antibody concentration was calculated from the optical density as a function of a standard curve of a recombinant human anti–SARS-CoV-2 spike S1 IgG antibody (GenScript, catalog no. A02038-1). The detection cutoff value was an optical density of 0.674 (*45*), which correlated to a concentration of 0.0009426984 μg/ml (fig. S17). Data in fig. S17 are means and 95% CIs for points. The corresponding trendline was estimated with a sigmoidal four-parameter logistic regression model. The figure shows the mean regression trend with a 95% confidence band.

### ECL of anti-S–IgG and ACE2 pseudo-neutralization (% inhibition)

SARS-CoV-2 variant cross-reactivity and the capacity of anti-S IgG to inhibit the binding of SARS-CoV-2 spike to ACE2 were characterized by ECL using the Meso Scale Discovery (MSD; Rockville, MD) V-PLEX SARS-CoV-2 IgG Panel 23 (catalog no. K15567U-2) and ACE2 Panel 23 (catalog no. K15570U-2) according to the manufacturer’s instructions. The MSD Panel 23 uses the World Health Organization International Standard (National Institute for Biological Standards and Control code: 20/136). Antibody concentrations were converted from MSD-assigned concentration units [arbitrary units (AU) per milliliter] to World Health Organization/NIBSC units in binding antibody units per milliliter by multiplying the values in arbitrary units per milliliter by 0.00901, which is the SARS-CoV-2 spike IgG conversion factor supplied by the manufacturer. On the basis of a comparison of IgG dynamics for the cohort conducted by running a subset of participant samples on both the ELISA and MSD platforms, we calculated that 1000 BAU/ml as measured on the MSD platform are approximately equal to 1 U (μg/ml) of antibody concentration as measured by the ELISA (fig. S18).

ACE2 pseudo-neutralization assays have been previously validated against live virus neutralization (*49*) and have been used by other studies (*50*, *51*). Only after dose 2 to 6-month post–dose 3 samples from whole blood were run on MSD assays because of sample volume requirements. Serum samples run on VPLEX SARS-CoV-2 Panel 23 were diluted by 20,000. Samples run on the corresponding pseudo-neutralization panel were diluted per the manufacturer’s instructions. All plates were prepared, and samples were run according to the kit instruction manual. A standard curve was run on each plate to allow for standardization across plates. We used data from the following SARS-CoV-2 variant antigens on panel 23: SARS-CoV-2 spike (ancestral), B.1.1.7 (Alpha), B.1.351 (Beta), P.1 (Gamma), B.1.617.2 (Delta), and B.1.1.529 (Omicron BA.1). ACE2 % inhibition was calculated using the MSD Workbench Application as follows

$$\%\;\mathrm{Inhibition}=\left(1-\frac{\text{Average sample ECL signal}}{\text{Average ECL signal of calibrator g}\;(\text{dilutent only})}\right)\times 100\%$$

### SARS-CoV-2 polymerase chain reaction, sequencing, and postvaccination infection surveillance

Participants submitted saliva using the OME-505 collection device (OMNIgene Oral, Ottawa, Canada) every 2 weeks from vaccination through 6 months after dose 3 to detect breakthrough SARS-CoV-2 infections. Viral RNA was extracted using a NucliSENS easyMag automated extraction system from 200 μl of saliva in stabilizing solution and eluted in a total volume of 50 μl. First-strand complementary DNA synthesis was performed from 5 μl of eluted RNA using SuperScript IV VILO Master Mix (Thermo Fisher Scientific). Positive specimens were then sequenced. Multiplex tiled amplicon libraries were prepared using the Midnight Panel and Rapid Barcoding Kit RBK-004 (Oxford Nanopore Technologies) using the previously published protocol (*52*). Twelve sample pooled libraries were sequenced on a GridION X5 nanopore sequencer using Flongle adapters. After sequencing, raw data were processed using interARTIC (*53*) to generate consensus sequences and variant calls. SARS-CoV-2 lineages were determined using these consensus sequences and the NextClade and Pangolin platforms (*54*, *55*).

The timing of breakthrough infections was used to assign the likeliest variant responsible for corresponding samples that could not be sequenced. This was accomplished by identifying the most common SARS-CoV-2 variant at the time of the breakthrough infection using data from CDC’s laboratory-based national variant estimates, available here: https://data.cdc.gov/Laboratory-Surveillance/SARS-CoV-2-Variant-Proportions/jr58-6ysp.

### Surveys

Standardized surveys that participants were asked to complete contained questions regarding their demographics, medical history, immune disorders, related comorbidities, immunoglobulin replacement regimens, medication usage, previous and known SARS-CoV-2 infections (whether symptomatic or not), and postvaccination adverse events (*56*) (as per the CDC’s V-safe system). Survey instruments can be found here: https://doi.org/10.5281/zenodo.7529357

### Statistical analyses

Medians were primarily used to characterize, compare, and visualize data, as some subgroup–by–time point strata had small sample sizes, rendering the arithmetic and geometric means suboptimal measures of central tendency. A Wilcoxon signed-rank test was used to compare median titer levels for IDP from the post–dose 3 and 6-month post–dose 3 paired samples. Fisher’s exact test was used to compare the percentage of seropositive IDP at the same time points. Wilcoxon rank sum tests were used to compare titer levels for HVs and IDP (considered collectively) across time points; pairwise Wilcoxon rank sum tests of each IDP group and HVs for each time point were conducted. All Wilcoxon rank sum tests used continuity corrections. For visualization purposes, figures were made with the ggplot2 R package (*57*), and data were pseudo-log_10_–transformed using the scales R package (*58*), where appropriate. Data points were jittered. Values below the limit of detection for the anti-S IgG ELISA (0.0009426984 μg/ml), anti-S IgG ECL assay (21.05 AU/ml as previously reported) (*59*), and ACE2 % inhibition (5% as determined by NIAID’s Laboratory of Virology) were substituted for the quotient of the limit of detection and the square root of 2. As some figures contained hundreds of data points for samples across SARS-CoV-2 variants, time, and subgroups, we opted to visualize data with the median and vertical bars typically shown in boxplots; we removed the boxes indicating the 25th and 75th percentiles to better see the underlying data.

Generalized additive mixed models (GAMMs) (*60*) were used to visualize the relationship between anti-S IgG antibody concentration and ACE2 % inhibition, both of which were measured with the MSD platform. GAMMs were built for variant-, time-, and subgroup-specific relationships. GAMMs used restricted maximum likelihood to estimate optimal smoothing parameters and thin-plate regression splines (*61*). Intraparticipant correlation across samples was accounted for using a random effect term at the individual level. The models used a quasibinomial distribution as the outcome variable was a percentage. GAMMs were constructed using the mgcv R package (*62*) and visualized using the ggplot2 R package by generalizing and correcting the plotting function found here: https://gist.github.com/richardbeare/b679c38dcb644ec50ea34ac061200504, which contained a simplified estimator for the SE. GAMM trend lines are visualized with their standard output, the mean, and 95% CI, unless noted otherwise.

The correlation between anti-S IgG antibody concentration and ACE2 inhibition was measured with the Kendall rank correlation coefficient using the confintr R package (*63*). Data were bootstrapped 9999 times at the individual level to account for intraparticipant correlation. Bias-corrected and accelerated 95% CIs were constructed from the bootstrap distributions. Comparisons of two correlation coefficients were facilitated by comparing the point estimate of one to the bootstrap distribution of the other and calculating one-sided empirical *P* values. Bootstrapping was efficiently performed using the dplyr (*64*), purrr (*65*), and coxed (*66*) R packages. Analyses were conducted using R version 4.1.1 (*67*).
