# Supplementary material for: Characterization of the antispike IgG immune response to COVID-19 vaccines in people with a wide variety of immunodeficiencies
Source: Sci Adv. 2023 Oct 12;9(41):eadh3150. doi: 10.1126/sciadv.adh3150 (PMC10569702; doi:10.1126/sciadv.adh3150)
Supplement: Supplementary file 1 — Figs. S1 to S18 Tables S1 to S11 [file sciadv.adh3150_sm.pdf]

Science Advances
AAAS

Supplementary Materials for

Characterization of the antispike IgG immune response to COVID-19 vaccines in people with a wide variety of immunodeficiencies

Mackenzie Zendt *et al.*

Corresponding author: Emily E. Ricotta, emily.ricotta@nih.gov

Sci. Adv. **9**, eadh3150 (2023)
DOI: 10.1126/sciadv.adh3150

This PDF file includes:

Figs. S1 to S18
Tables S1 to S11

SUPPLEMENTAL FIGURES

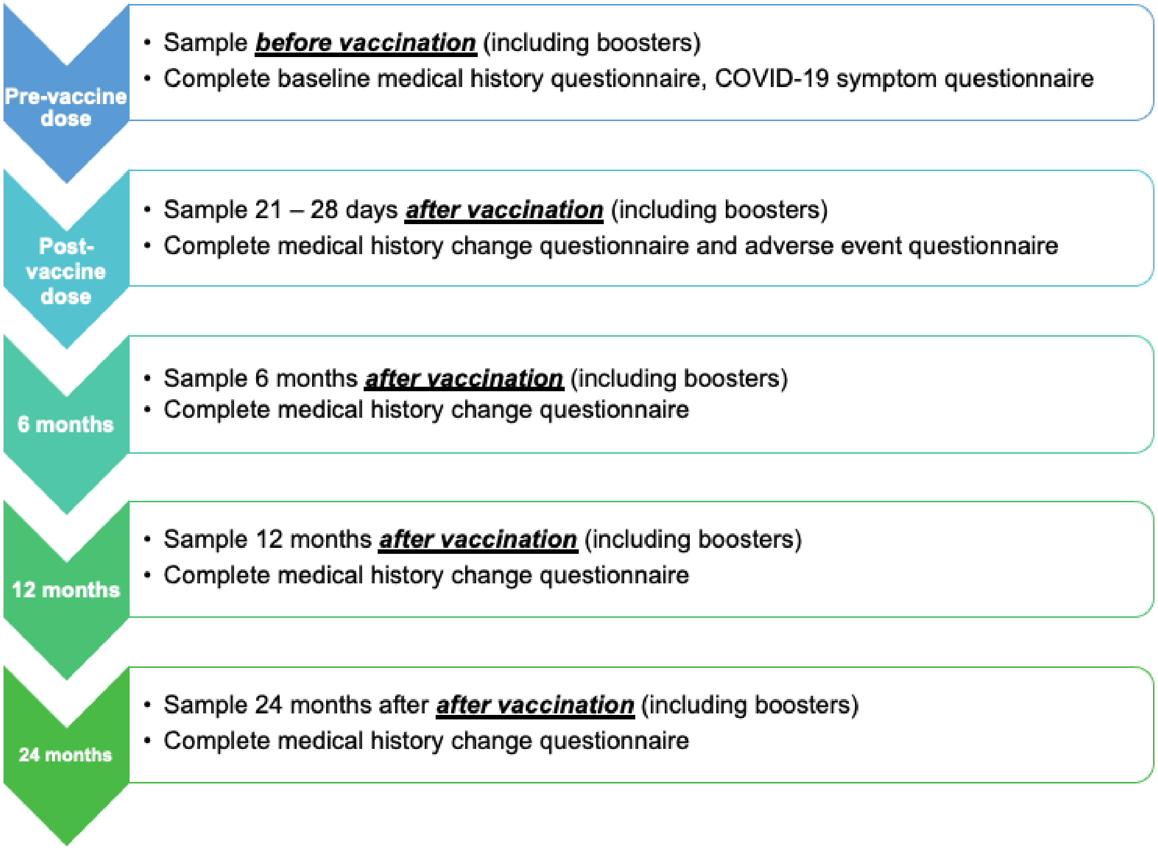

Figure S1. Sample collection timeline.

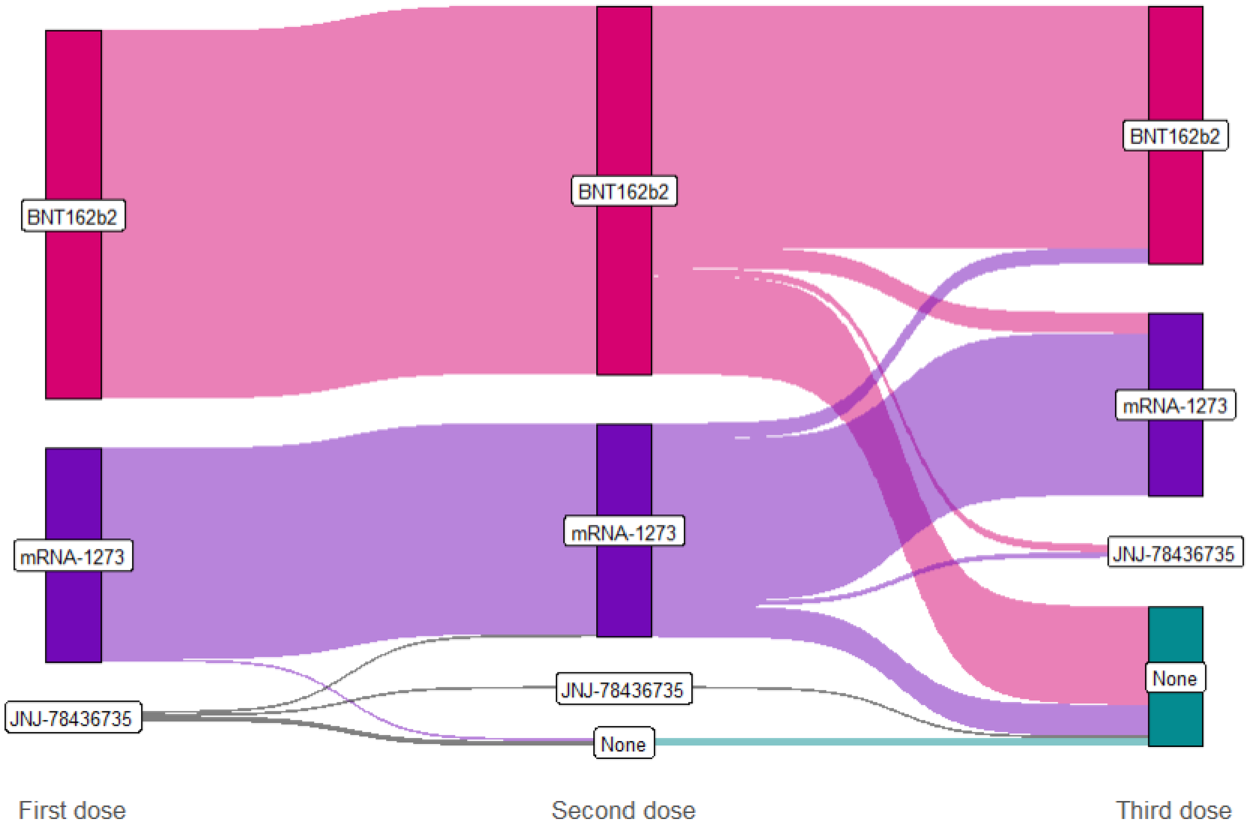

Figure S2. Sankey plot of vaccine brand received for all 230 participants during the study period.

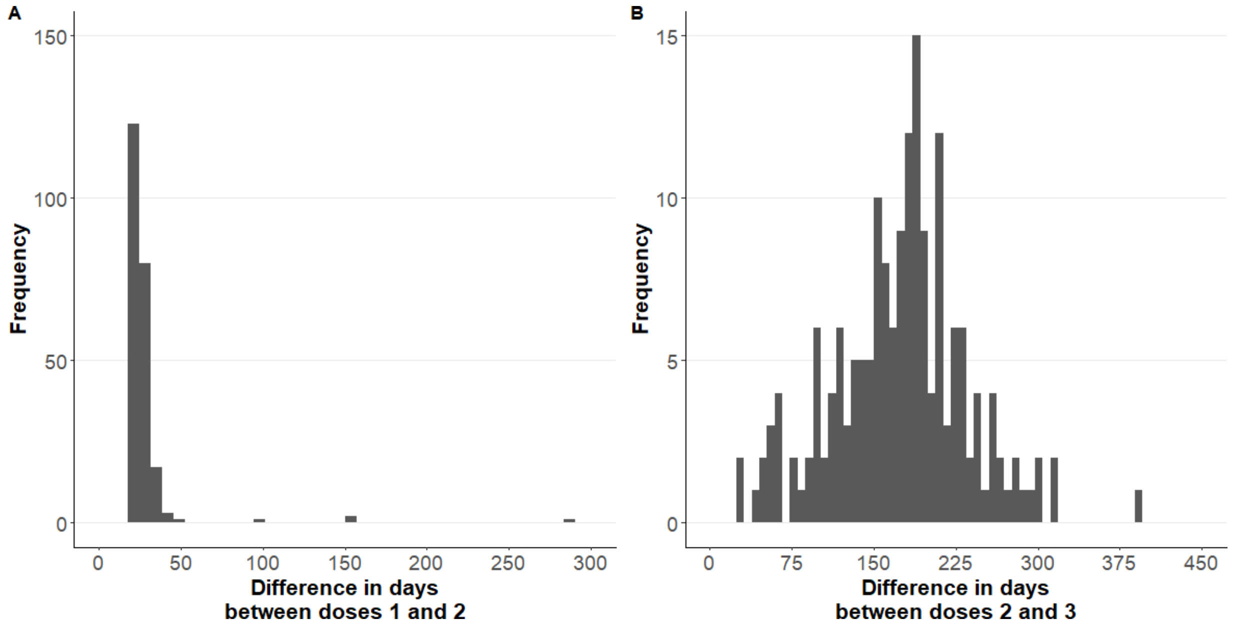

Figure S3. Histograms of the number of days between vaccinations. The histogram bandwidth is same across two panels. **A)** Distribution of the number of days between dose 1 and dose 2. The mean is 27.5 days and the median is 22 days. **B)** Distribution of the number of days between dose 2 and dose 3. The mean is 174.5 days and the median is 180.5 days.

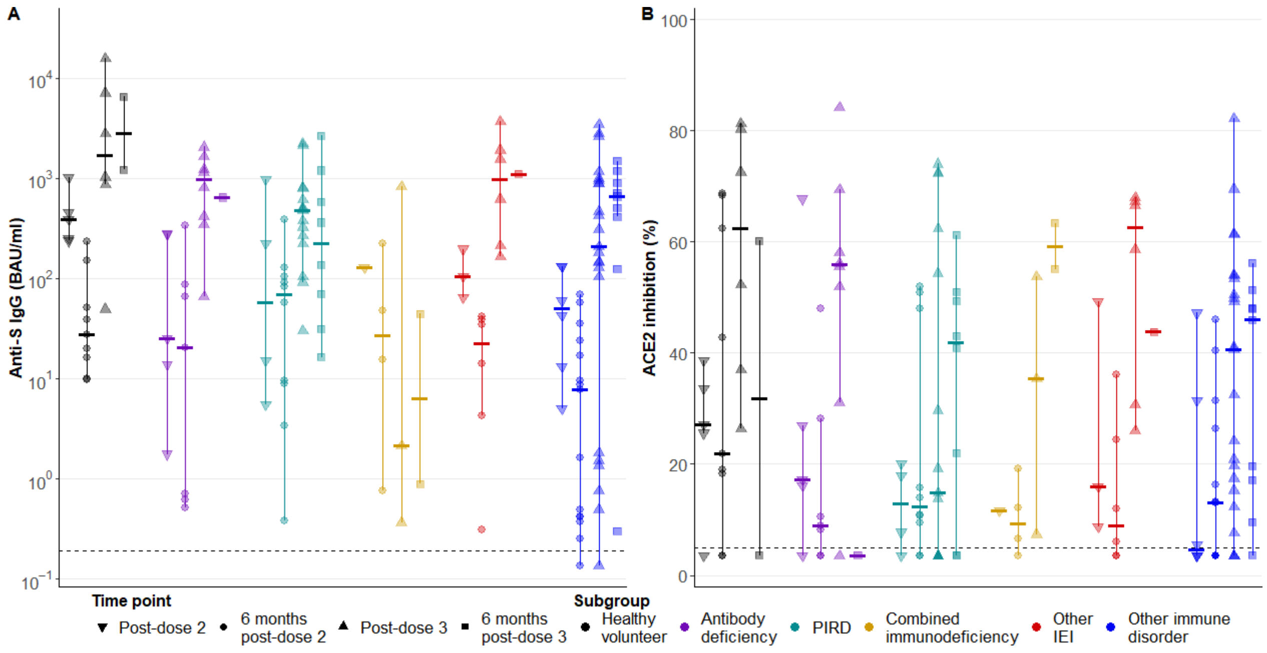

Figure S4. Anti-S IgG antibody concentration and ACE2 pseudo-neutralization (% inhibition) for the Omicron BA.1 variant. Vertical bars extend from the median (horizontal bars) to the end of a standard boxplot's whiskers. Data shown are measured by electrochemiluminescence. Samples from participants with evidence of prior SARS-CoV-2 infection, those on AZD7442 (tixagevimab- cilgavimab, AstraZeneca), and post-breakthrough infection were removed to quantify the impact of the vaccines against an immunologically naïve background. The dashed line shows the assay limit of detection. Three outliers were removed from this chart for visualization purposes. **A)** IgG concentration against the Omicron BA.1 variant across timepoints and immunological subgroups. Comparable data for the ancestral strain is shown in Figure 2A. **B)** ACE2 inhibition data against the Omicron BA.1 variant across time and immunological subgroups. Comparable data for the ancestral strain is shown in Figure 2B.

Abbreviations: ACE2, angiotensin converting enzyme 2; IEI, inborn error of immunity; IgG, immunoglobulin G; IgRT, immunoglobulin replacement therapy; SARS-CoV-2, severe acute respiratory syndrome coronavirus 2; PIRD, primary immune regulatory disorders

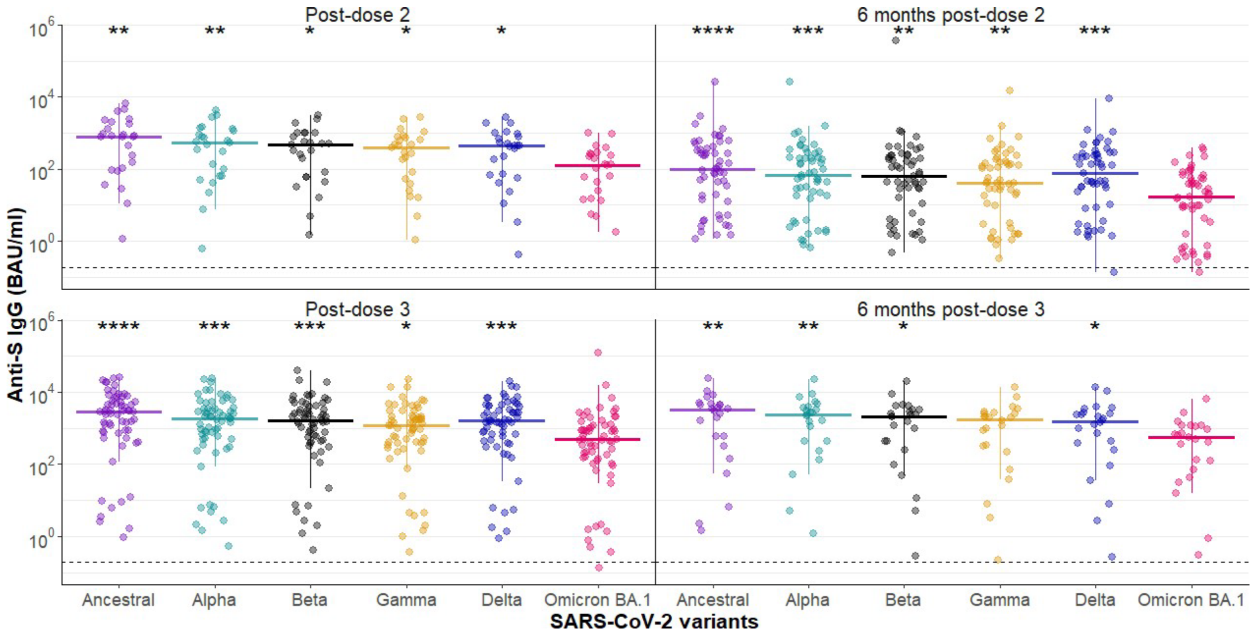

Figure S5. Anti-S IgG antibody concentration by SARS-CoV-2 variant and timepoint. Vertical bars extend from the median (horizontal bars) to the end of a standard boxplot's whiskers. Data shown are measured by electrochemiluminescence. Samples corresponding to participants with evidence of prior SARS-CoV-2 infectionthose on AZD7442 (tixagevimab-cilgavimab, AstraZeneca), and timepoint post- breakthrough infections were removed to quantify the impact of the vaccines against an immunologically naïve background. The Wilcoxon rank-sum test was used to assess pairwise differences in medians, with all within-timepoint comparisons against Omicron BA.1. By convention, * = $p \leq 0.05$, ** = $p \leq 0.01$, *** = $p \leq 0.001$, and **** = $p \leq 0.0001$. The dashed line indicates the assay limit of detection.

Abbreviations: IgG, immunoglobulin G; SARS-CoV-2, severe acute respiratory syndrome coronavirus 2

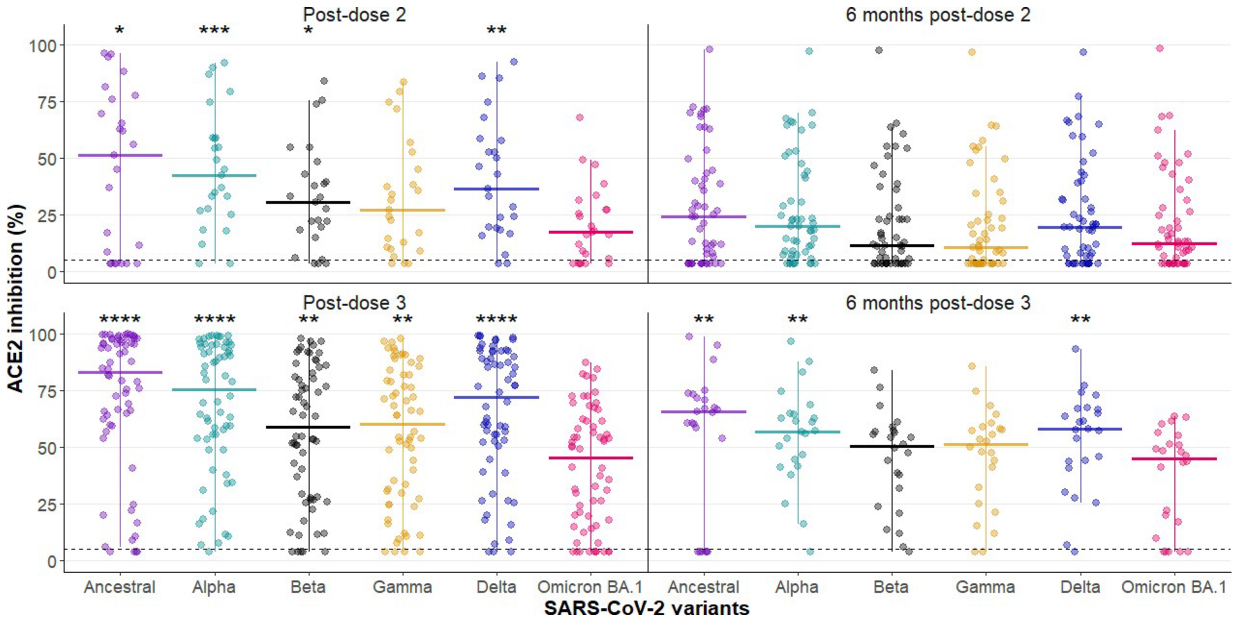

Figure S6. ACE2 pseudo-neutralization (% inhibition) by SARS-CoV-2 variant and timepoint. Vertical bars extend from the median (horizontal bars) to the end of a standard boxplot's whiskers. Data shown are measured by electrochemiluminescence. Samples corresponding to participants with evidence of prior SARS-CoV-2 infectionthose on AZD7442 (tixagevimab-cilgavimab, AstraZeneca), and timepoint post-breakthrough infections were removed to quantify the impact of the vaccines against an immunologically naïve background. The Wilcoxon rank-sum test was used to assess pairwise differences in medians, with all comparisons against Omicron BA.1. By convention, * = $p \leq 0.05$, ** = $p \leq 0.01$, *** = $p \leq 0.001$, and **** = $p \leq 0.0001$. The dashed line indicates the assay limit of detection.

Abbreviations: ACE2, angiotensin converting enzyme 2; SARS-CoV-2, severe acute respiratory syndrome coronavirus 2

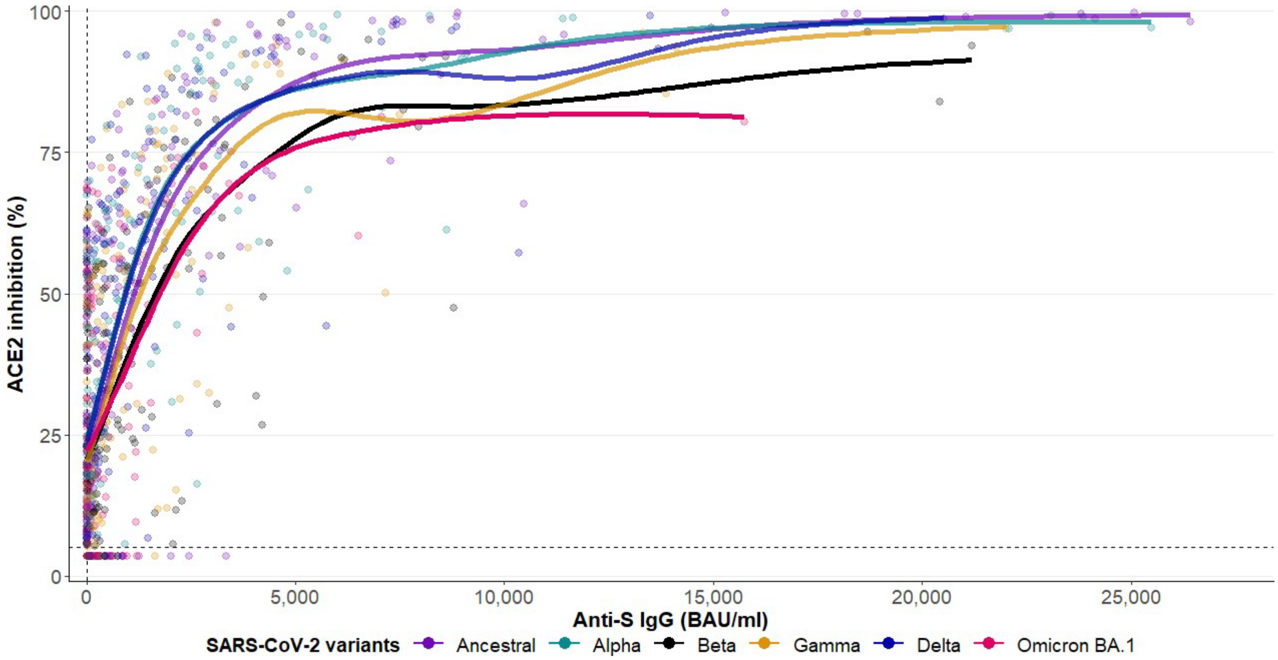

Figure S7. GAMM trendlines of ACE2 pseudo-neutralization capacity (% inhibition) as functions of anti-S IgG concentration by SARS-CoV-2 variant. Points represent anti-S IgG concentration and ACE2 % inhibition values for each SARS-CoV-2 variant of concern. Variant-specific GAMMs are used to show the mean variant-specific trend. For statistical convergence, the model aggregates over the six immunological subgroups (*i.e.*, healthy volunteers, antibody deficiencies, PIRD, combined immunodeficiencies, other IEI, other immune disorders) and four timepoints (*i.e.*, post-dose 2, six months post-dose 2, post-dose 3, and six months post-dose 3). The mixed model incorporates a random effect at the individual level to account for intra-person correlation. Samples corresponding to participants with evidence of prior SARS-CoV-2 infection, those on AZD7442 (tixagevimab-cilgavimab, AstraZeneca), and timepoints post-breakthrough infections were removed to quantify the impact of the vaccines against an immunologically naïve background. This figure plots and models the raw values shown in Figure 2C rather than showing the IgG concentration data on a log10 scale. Dashed lines correspond to assay limits of detection. Three outliers were removed from this chart for visualization purposes.

Abbreviations: ACE2, angiotensin converting enzyme 2; GAMM, generalized additive mixed model; IgG, immunoglobulin G; SARS-CoV-2, severe acute respiratory syndrome coronavirus 2

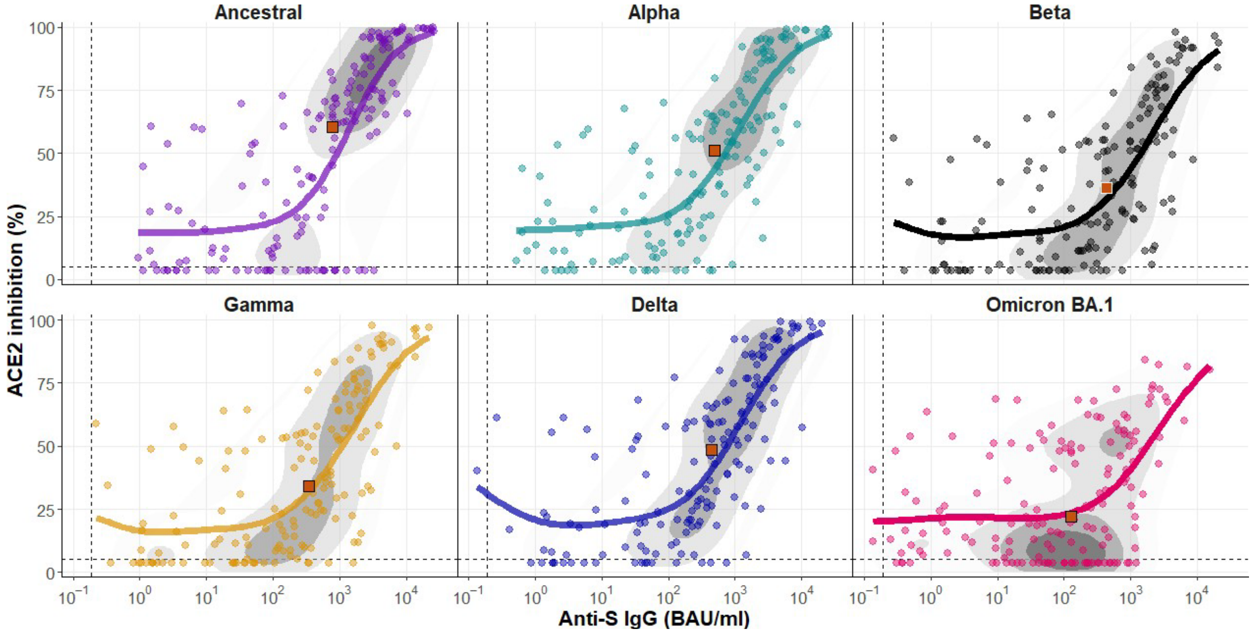

Figure S8. GAMM trendlines of ACE2 pseudo-neutralization capacity (% inhibition) as functions of anti-S IgG antibody concentration SARS-CoV-2 variants with accompanying contours from a two-dimensional kernel density estimation. Points represent anti-S IgG concentration and ACE2 % inhibition values for each SARS-CoV-2 variant of concern. A two-dimensional kernel density estimation was used, with the same settings across variants to generate contours, which show the density of the points. Darker contours indicate a higher concentration of points, and vice versa. For plotting purposes, anti-S IgG concentration values (on the x-axis) were transformed using a base-10 logarithm since the kernel density algorithm requires a reasonably regular grid to generate contour plots. A GAMM, which captures non-linear trend, shows the mean trend and is superimposed on the kernel density. For statistical convergence, the model aggregates over the six immunological subgroups (*i.e.*, healthy volunteers, antibody deficiencies, PIRD, combined immunodeficiencies, other IEI, other immune disorders) and four timepoints (*i.e.*, post-dose 2, six months post-dose 2, post-dose 3, and six months post-dose 3). These trendlines are the same as those in Figure 2C. The mixed model incorporates a random effect at the individual level to account for intra-person correlation. The confidence band is not shown to better visualize the underlying kernel density-based contours. The orange squares indicate the median values for anti-S IgG concentration and ACE2 inhibition. Samples corresponding to participants with evidence of prior SARS-CoV-2 infection, those on AZD7442 (tixagevimab-cilgavimab, AstraZeneca), and timepoints post-breakthrough infections were removed to quantify the impact of the vaccines against an immunologically naïve background. Dashed lines indicate the assay limits of detection. Three outliers were removed from this chart for visualization purposes.

Abbreviations: ACE2, angiotensin converting enzyme 2; GAMM, generalized additive mixed model; IgG, immunoglobulin G; SARS-CoV-2, severe acute respiratory syndrome coronavirus 2

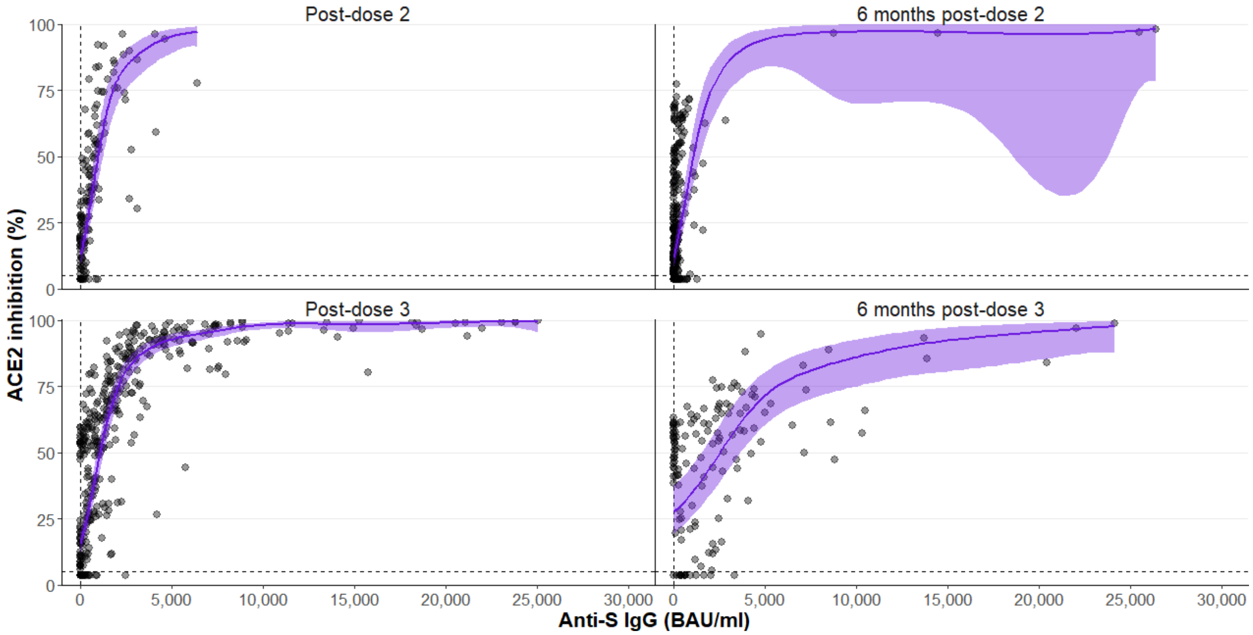

Figure S9. GAMM trendlines of ACE2 pseudo-neutralization capacity (% inhibition) as functions of anti-S IgG antibody concentrations by sample timepoint. Each trend line is shown with a corresponding 95% confidence band. For statistical convergence, the model aggregates over the six immunological subgroups (*i.e.*, healthy volunteers, antibody deficiencies, PIRD, combined immunodeficiencies, other IEI, other immune disorders) and six SARS-COV-2 variants (*i.e.*, Ancestral, Alpha, Beta, Gamma, Delta, and Omicron BA.1). Individual values are shown as dots. The mixed model incorporates a random effect at the individual level to account for intra-person correlation. Samples corresponding to participants with evidence of prior SARS-CoV-2 infection, those on AZD7442 (tixagevimab- cilgavimab, AstraZeneca), and timepoints post-breakthrough infections were removed to quantify the impact of the vaccines against an immunologically naïve background. The dashed lines show the assay limits of detection. Three outliers were removed from this chart for visualization purposes.

Abbreviations: ACE2, angiotensin converting enzyme 2; GAMM, generalized additive mixed model; IgG, immunoglobulin G; SARS-CoV-2, severe acute respiratory syndrome

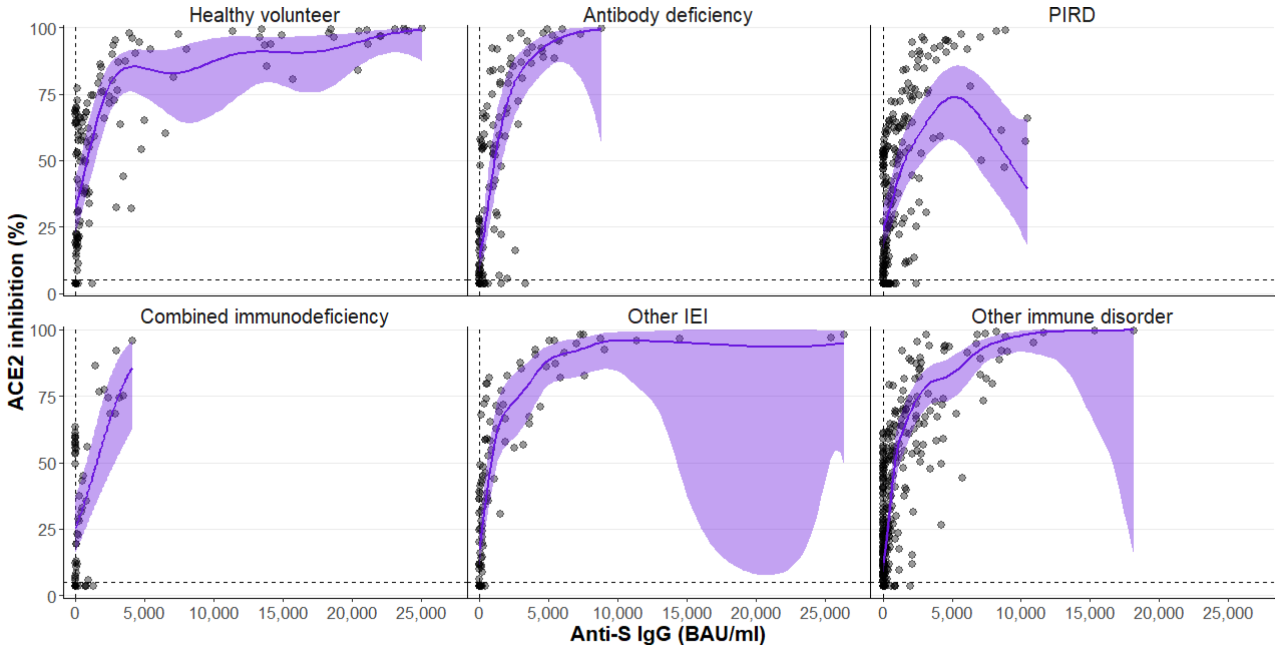

Figure S10. GAMM trendlines of ACE2 pseudo-neutralization capacity (% inhibition) as functions of IgG anti-S IgG antibody concentrations by immunological subgroup. Each trend line is shown with a corresponding 95% confidence band. For statistical convergence, the model aggregates over four timepoints (*i.e.*, post-dose 2, six months post-dose 2, post-dose 3, and six months post-dose 3) and six SARS-CoV-2 variants (*i.e.*, Ancestral, Alpha, Beta, Gamma, Delta, and Omicron BA.1). Individual values are shown as dots. The mixed model incorporates a random effect at the individual level to account for intra-person correlation. Samples corresponding to participants with evidence of prior SARS-CoV-2 infection, those on AZD7442 (tixagevimab-cilgavimab, AstraZeneca), and timepoints collected after breakthrough infections were removed to quantify the impact of the vaccines against an immunologically naïve background. The dashed lines show the assay limits of detection. Three outliers were removed from this chart for visualization purposes.

Abbreviations: ACE2, angiotensin converting enzyme 2; GAMM, generalized additive mixed model; IEI, inborn errors of immunity; IgG, immunoglobulin G; SARS-CoV-2, severe acute respiratory syndrome; PIRD, primary immune regulatory disorders

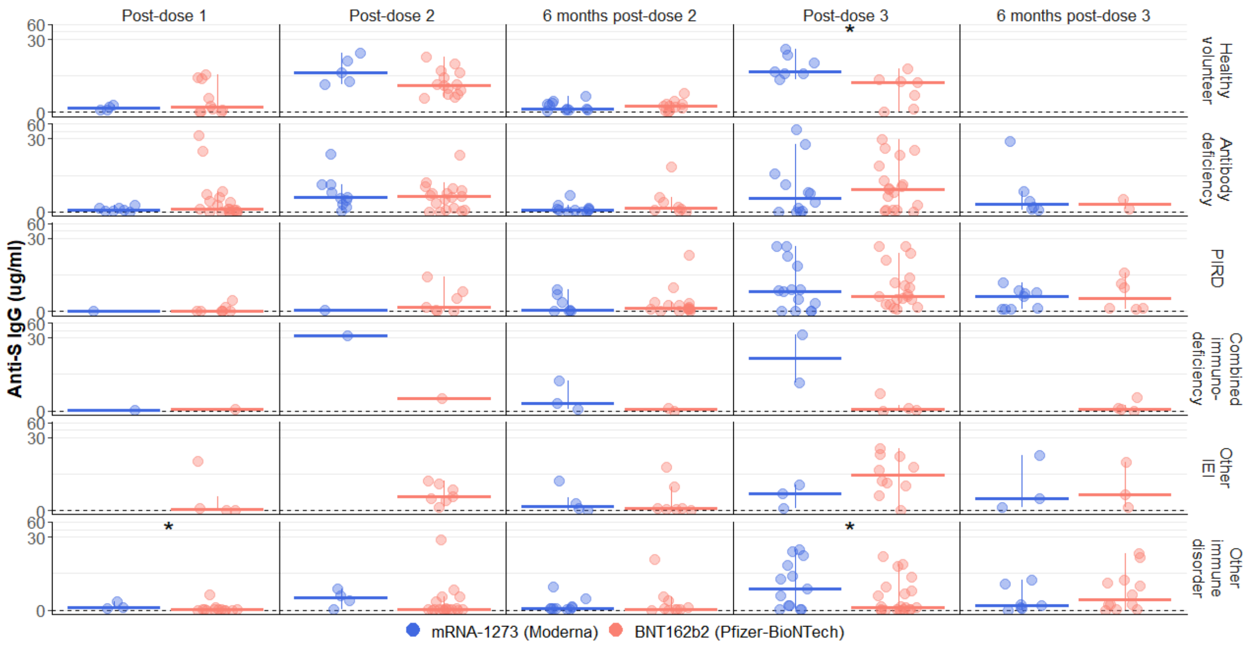

Figure S11. Anti-S IgG antibody titers by vaccine brand across timepoints and immunological subgroups. Vertical bars extend from the median (horizontal bars) to the end of a standard boxplot's whiskers. The Wilcoxon rank-sum test was used to evaluate differences in titer levels across the multiple vaccine brands. Only participants who received the same vaccine brand across all doses were included. Participants who received JNJ-78436735 (Janssen) (n=9) at any point were excluded. By convention, * = $p \leq 0.05$, ** = $p \leq 0.01$, *** = $p \leq 0.001$, and **** = $p \leq 0.0001$. The dashed line indicates the assay limit of detection.

Abbreviations: IgG, immunoglobulin G

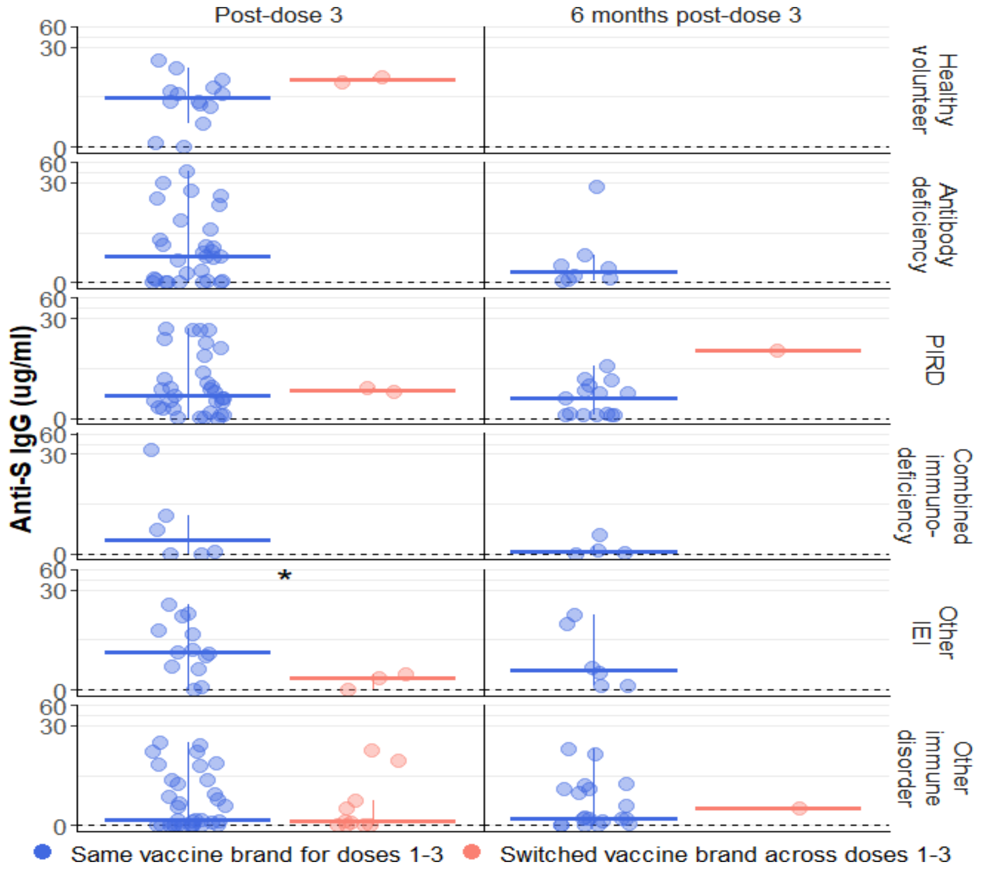

Figure S12. Anti-S IgG titers of participants by timepoint, immunological subgroup, and vaccine switching status. Vertical bars extend from the median (horizontal bars) to the end of a standard boxplot's whiskers. The Wilcoxon rank-sum test was used to evaluate differences in median titer levels across vaccine brands by time. Only the post-dose 3 and six months post-dose 3 timepoints are examined because almost all participants received the mRNA-based vaccines, which have a two-dose primary series for immunocompetent participants, and no participant who received either BNT162b2 (Pfizer-BioNTech) or mRNA-1273 (Moderna) at dose 1 switched vaccine brands at dose 2. As a result, only individuals with 3 doses are considered. While most participants switched between mRNA-based vaccines at dose 3, this analysis includes data for individuals who switched from BNT162b2 or mRNA-1273 to JNJ-78436735 (Janssen) for the third dose. However, removing the JNJ-78436735 recipients did not alter the conclusions. No data for individuals who received JNJ-78436735 for doses 1 or 2 are shown as no such participant received a third dose. By convention, * = $p \leq 0.05$, ** = $p \leq 0.01$, *** = $p \leq 0.001$, and **** = $p \leq 0.0001$. The dashed line indicates the limit of detection.

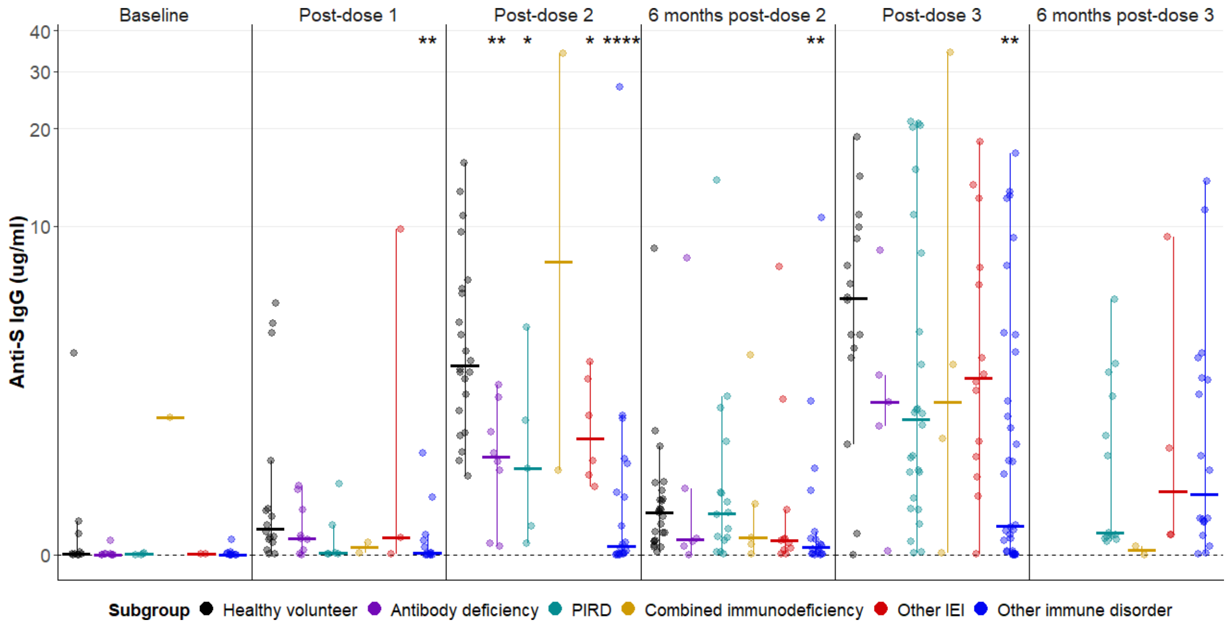

Figure S13. Anti-S IgG titers of study participants not receiving IgRT. Vertical bars extend from the median (horizontal bars) to the end of a standard boxplot's whiskers. Data in the figure are restricted to participants not receiving IgRT during the study period and those who did not report whether they received IgRT (as in Figure 3a). However, restricting to only those not receiving IgRT led to very similar results. Pairwise comparisons between the HVs control group and each IDP subgroup at each timepoint were made using the Wilcoxon rank-sum text. No comparisons were possible at the six months post-dose 3 timepoint as healthy volunteers were not eligible for a third dose until late in the study period, per vaccination guidelines from the Centers for Disease Control and Prevention. By convention, * = $p \leq 0.05$, ** = $p \leq 0.01$, *** = $p \leq 0.001$, and **** = $p \leq 0.0001$. The dashed line indicates the assay limit of detection.

Abbreviations: HVs, healthy volunteers; IDP; immune-deficient/disordered person; IEI, inborn errors of immunity; IgG, immunoglobulin G; IgRT, immunoglobulin replacement therapy; PIRD, primary immune regulatory disorders

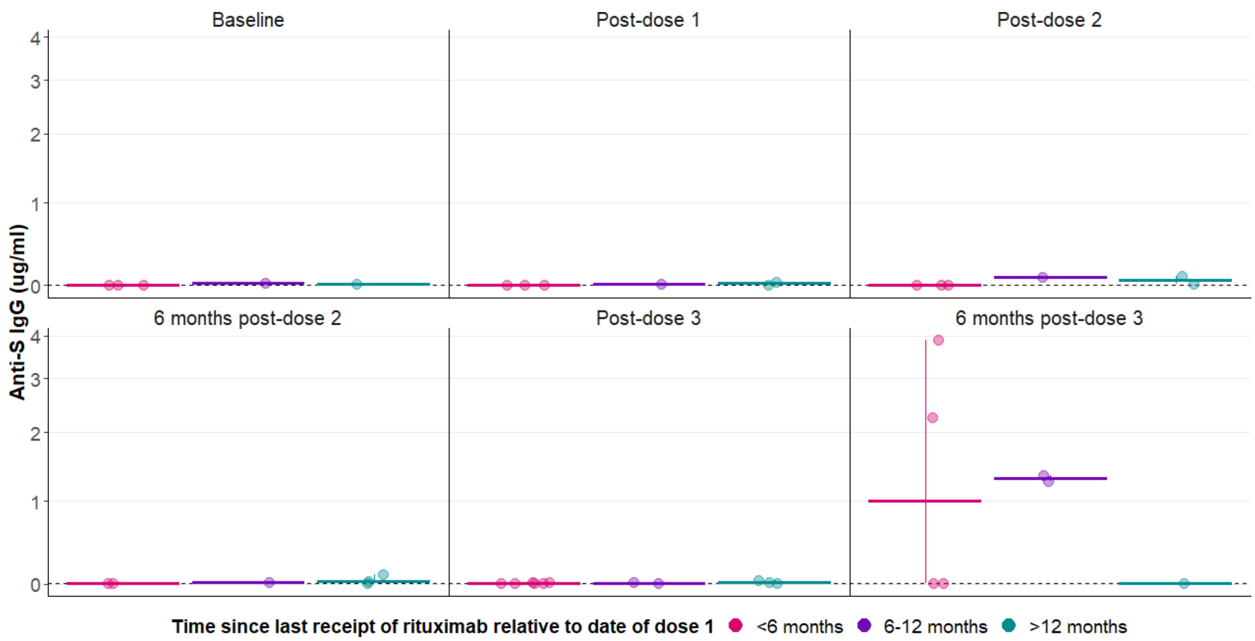

Figure S14. Anti-S IgG titers of participants who received rituximab prior to vaccine dose 1. Vertical bars extend from the median (horizontal bars) to the end of a standard boxplot's whiskers. Data for the 15 participants who received rituximab before dose 1 are shown by timepoint. All data are grouped by time from last receipt of rituximab relative to the date of dose 1. Three of the four participants whose anti-S IgG titers are >1ug/ml at six months post-dose 3 received AZD7442 (tixagevimab-cilgavimab, AstraZeneca) before their six month post-dose 3 sample. The dashed line indicates the assay limit of detection.

Abbreviations: IgG, immunoglobulin G

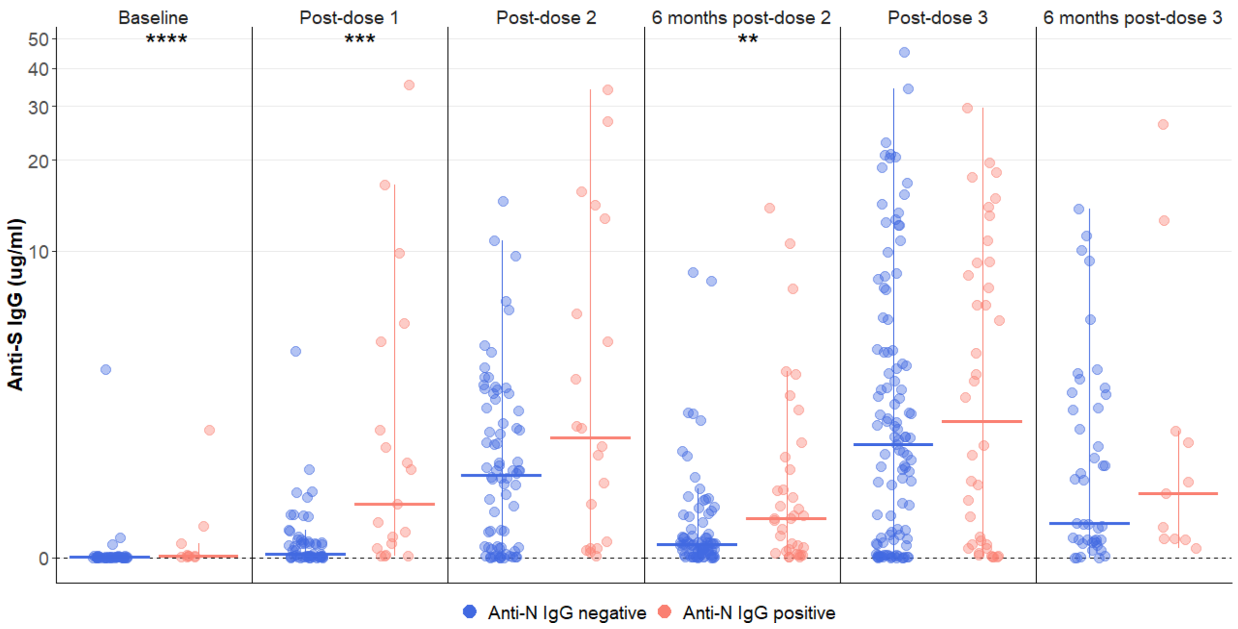

Figure S15. Anti-S IgG titers by nucleocapsid-positive status by time. Vertical bars extend from the median (horizontal bars) to the end of a standard boxplot's whiskers. The Wilcoxon rank-sum test was used for all comparisons. In all cases, missing values were coded as "No." The figure shows all participants in the study, including IDP on IgRT. Nucleocapsid positivity was determined by ELISA and assessed at each timepoint. A lack of asterisks indicates a non-significant comparison. By convention, * = $p \leq 0.05$, ** = $p \leq 0.01$, *** = $p \leq 0.001$, and **** = $p \leq 0.0001$. The dashed line indicates the assay limit of detection.

Abbreviations: ELISA, enzyme-linked immunosorbent assays; IDP, immune-deficient/disordered people; IgG, immunoglobulin G; IgRT, immunoglobulin replacement therapy

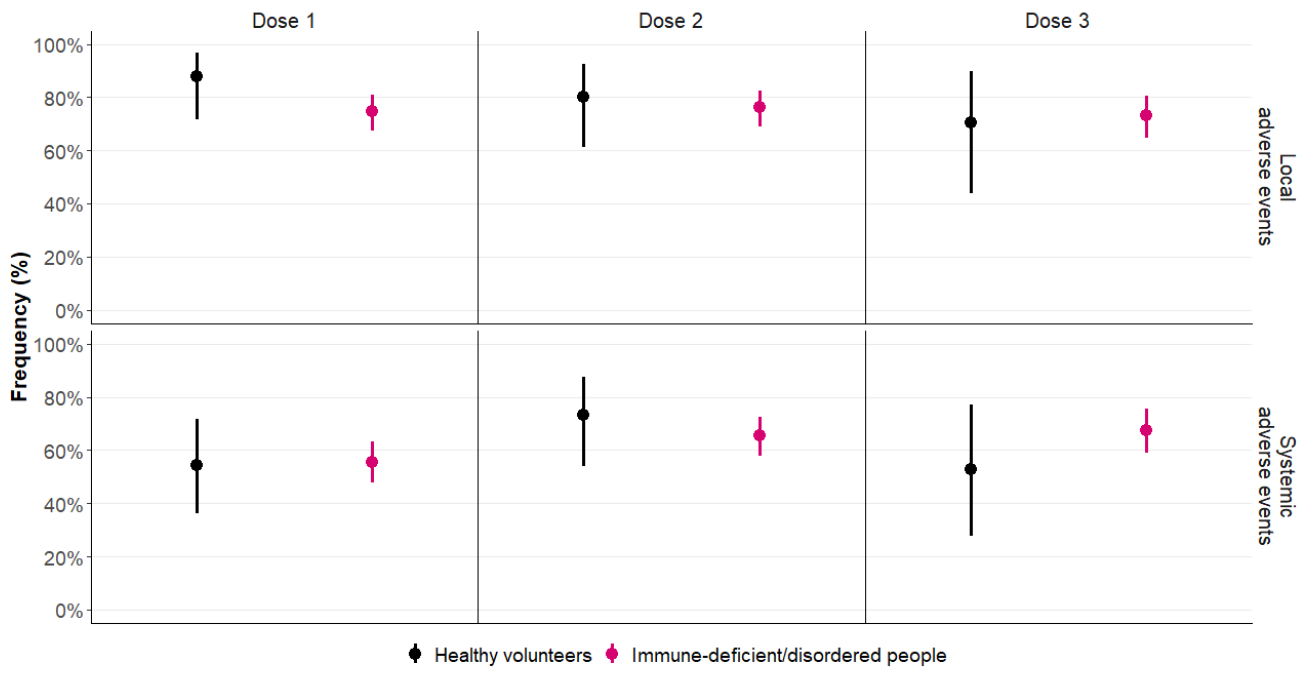

Figure S16. Proportion of vaccine-induced adverse events in IDP and HVs. There was no significant difference in frequency of local or systemic events between IDP or HVs. Means and 95% Clopper-Pearson confidence intervals are shown. Local adverse events were defined as the presence of pain, redness, itching, and swelling at the injection site. Systemic adverse events were defined as the presence of chills, headache, joint pain, muscle aches, fatigue, nausea, vomiting, diarrhea, abdominal pain, itching in the mouth, body rash, heart palpitations, or dizziness.

Abbreviations: IDP, immune-deficient/disordered people; HVs, healthy volunteers

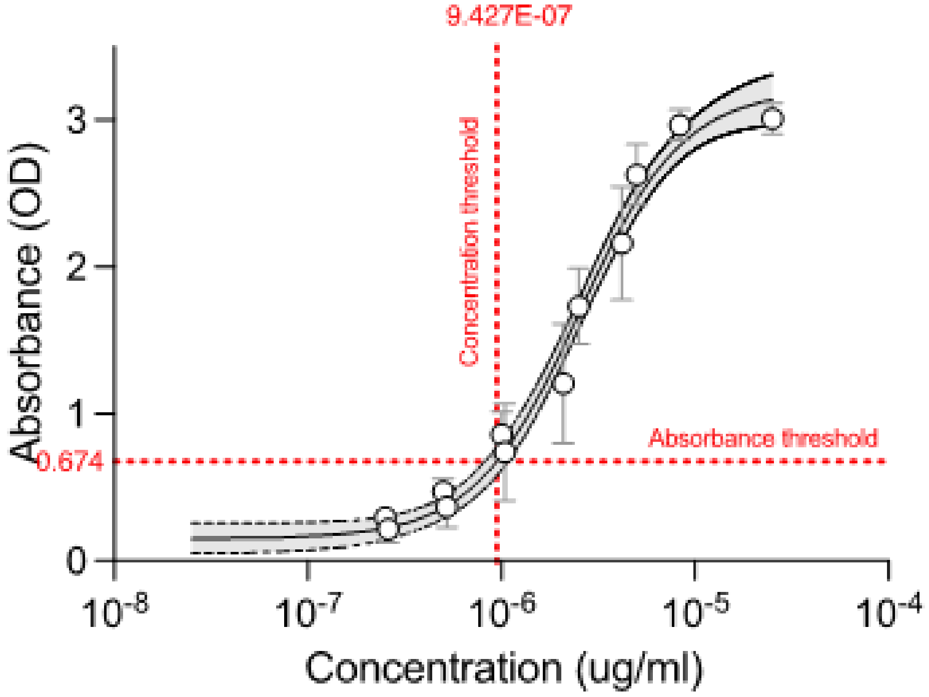

Figure S17. The association between anti-spike IgG concentration and optical density. Data points in the figure are means and related 95% confidence intervals. The trend line for all the data was estimated with a sigmoidal four-parameter logistic regression model. The mean regression trend and a 95% confidence band is shown.

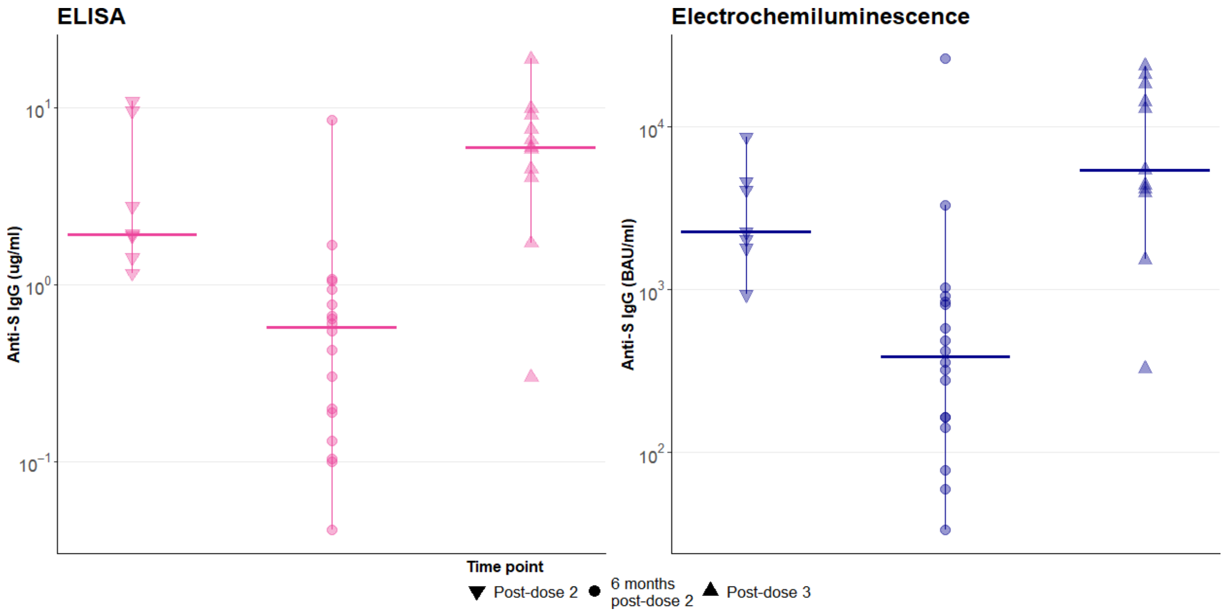

Figure S18. Cross-validation of anti-S IgG by ELISA and electrochemiluminescence. Vertical bars extend from the median (horizontal bars) to the end of a standard boxplot's whiskers. Data is shown for HVs that supplied samples at the post-dose 2, 6 months post-dose 2, and post-dose 3 timepoints and for which there was enough sample volume to analyze by both ELISA and electrochemiluminescence.

Abbreviations: ELISA, enzyme-linked immunosorbent assays; IgG, immunoglobulin G; HVs, healthy volunteers

SUPPLEMENTAL TABLES

Table S1. Specific immunodeficiencies of IDP participants.

**Immunological subgroup**	**Specific Immunodeficiency**	**N**
Antibody deficiency	Common variable immunodeficiency	31
Antibody deficiency	Hypogammaglobulinemia	8
Antibody deficiency	NFkB1 haploinsufficiency	1
Antibody deficiency	Specific antibody deficiency	10
Antibody deficiency	X-linked agammaglobulinemia	2
Combined immunodeficiency	CD4 lymphocytopenia	4
Combined immunodeficiency	DiGeorge syndrome	1
Combined immunodeficiency	DOCK8 deficiency	1
Combined immunodeficiency	NEMO deficiency	1
Combined immunodeficiency	SASH3	1
Combined immunodeficiency	Severe combined immune deficiency	1
Primary immune regulatory disorders	Autoimmune lymphoproliferative syndrome	1
Primary immune regulatory disorders	Activated PI3K delta syndrome	6
Primary immune regulatory disorders	Autoimmune polyendocrinopathy-candidiasis-ecto-dermal dystrophy	16
Primary immune regulatory disorders	CTLA4 deficiency	3
Primary immune regulatory disorders	STAT1 autosomal dominant loss of function	1
Primary immune regulatory disorders	STAT1 gain of function	2
Primary immune regulatory disorders	STAT3 dominant-negative	17
Other inborn errors of immunity	Autoantibodies to GM-CSF	3
Other inborn errors of immunity	Autoantibodies to IFN-γ	1
Other inborn errors of immunity	C4 complement deficiency	1
Other inborn errors of immunity	Chronic granulomatous disease	5
Other inborn errors of immunity	COPA mutation	1
Other inborn errors of immunity	DADA2 deficiency	4
Other inborn errors of immunity	GATA2 haploinsufficiency	1
Other inborn errors of immunity	Hereditary angioedema	1
Other inborn errors of immunity	Autosomal dominant IFN-γ receptor deficiency	1
Other inborn errors of immunity	IRAK4 deficiency	1
Other inborn errors of immunity	Myhre syndrome	2

Other inborn errors of immunity	SCT/Rac2 exon 3	1
Other inborn errors of immunity	Thymoma/Good syndrome	4
Other immune disorders	Autoimmune	20
Other immune disorders	Bone marrow transplant	2
Other immune disorders	Severe combined immune deficiency post-bone marrow transplant/gene therapy	4
Other immune disorders	Chronic lymphocytic leukemia	13
Other immune disorders	Chronic myeloid leukemia	1
Other immune disorders	Hairy cell leukemia	1
Other immune disorders	Idiopathic membranous nephropathy	1
Other immune disorders	Lymphoma	1
Other immune disorders	Merkel cell carcinoma	1
Other immune disorders	Multiple myeloma	2
Other immune disorders	Myelofibrosis	1
Other immune disorders	Non-Hodgkin's lymphoma	4
Other immune disorders	T cell large granular lymphocytic leukemia	1
Other immune disorders	Solid organ transplant	7
Other immune disorders	Waldenström macroglobulinemia	3

Abbreviations: COPA, coatomer protein, subunit alpha; CTLA4, cytotoxic T-lymphocyte associated protein 4 ; DADA2, deficiency of the enzyme ADA2 (adenosine deaminase 2); DOCK8, dedicator of cytokinesis 8; IDP, immune-deficient/disordered people; IFN-γ, interferon gamma; IRAK4, interleukin 1 receptor associated kinase 4; GATA2, GATA-binding factor 2; GM-CSF, granulocyte macrophage-colony stimulating factor; NEMO, nuclear factor-kappa B essential modulator; NFB1, nuclear factor kappa B subunit 1; PI3K, phosphoinositide 3-kinases ; SASH3, SAM and SH3 domain containing 3; STAT 1/3, signal transducer and activator of transcription 1/3

Table S2. Number of samples available by timepoint and immunological subgroup.

Timepoint	Immunological subgroup	N
Baseline	Healthy volunteers	10
	Antibody deficiency	21
	PIRD	7
	Combined immunodeficiency	1
	Other IEI	2
	Other immune disorder	14
Post-dose 1	Healthy volunteers	16
	Antibody deficiency	29
	PIRD	9
	Combined immunodeficiency	2
	Other IEI	4
	Other immune disorder	16
Post-dose 2	Healthy volunteers	22
	Antibody deficiency	30
	PIRD	8
	Combined immunodeficiency	2
	Other IEI	7
	Other immune disorder	25
6 months post-dose 2	Healthy volunteers	25
	Antibody deficiency	21
	PIRD	24
	Combined immunodeficiency	5
	Other IEI	14
	Other immune disorder	24
Post-dose 3	Healthy volunteers	16
	Antibody deficiency	30
	PIRD	35
	Combined immunodeficiency	6
	Other IEI	16
	Other immune disorder	44
6 months post-dose 3	Healthy volunteers	0
	Antibody deficiency	8
	PIRD	16

	Combined immunodeficiency	4
	Other IEI	6
	Other immune disorder	20

Abbreviations: IEI, inborn errors of immunity; PIRD, primary immune regulatory disorder

Table S3. Vaccine brand received by immune status for all participants.

Immunological subgroup	Vaccine 1	Vaccine 2	Vaccine 3	N
Healthy volunteers	BNT162b2	BNT162b2	BNT162b2	7
	BNT162b2	BNT162b2	mRNA-1273	2
	BNT162b2	BNT162b2	None	11
	mRNA-1273	mRNA-1273	mRNA-1273	11
	mRNA-1273	mRNA-1273	None	2
	JNJ-78436735	mRNA-1273	None	1
	JNJ-78436735	None	None	1
Immune-deficient/disordered people	BNT162b2	BNT162b2	BNT162b2	87
	BNT162b2	BNT162b2	mRNA-1273	6
	BNT162b2	BNT162b2	JNJ-78436735	3
	BNT162b2	BNT162b2	None	27
	mRNA-1273	mRNA-1273	BNT162b2	6
	mRNA-1273	mRNA-1273	mRNA-1273	52
	mRNA-1273	mRNA-1273	JNJ-78436735	2
	mRNA-1273	mRNA-1273	None	9
	mRNA-1273	None	None	1
	JNJ-78436735	JNJ-78436735	None	1
	JNJ-78436735	None	None	1

Table S4. Median anti-S IgG antibody titers of IDP subgroups as a percentage of median antibody titers of HVs.[1-3]

Timepoint	HVs	All IDP[4]		Antibody deficiency	Primary immune regulatory disorder	Combined immuno-deficiency	Other inborn errors of immunity	Other immune disorder
Post-dose 1	100.0	14.1		58.1	5.5	NR	NR	3.8
Post-dose 2	100.0	24.5		40.0	8.0	NR	40.2	3.9
6 months post-dose 2	100.0	33.3		34.4	40.1	39.9	29.8	18.1
Post-dose 3	100.0	29.0		35.3	35.4	17.1	50.4	6.3

[1] Percentages are calculated row-wise relative to the healthy volunteers.
[2] Data with fewer than five IDP at a given timepoint are not reported due to a lack of statistical confidence in the IDP-specific median.
[3] Data are not presented for the six months post-dose 3 timepoint as healthy volunteers were not eligible for a third dose during the study period, per vaccination guidelines from the Centers for Disease Control and Prevention.
[4] This column includes all IDP in the study.

Abbreviations: HVs, healthy volunteers; IDP, immune-deficient/disordered people; IgG; immunoglobulin G; NR, not reported

Table S5. Median anti-S IgG antibody titers (ug/ml) by immunological subgroup.[1,2]

Timepoint	HVss	All IDP[3]		Antibody deficiency	Primary immune regulatory disorder	Combined immuno-deficiency	Other inborn errors of immunity	Other immune disorder
Post-dose 1	0.365	0.052		0.212	0.020	NR	NR	0.014
Post-dose 2	3.525	0.863		1.408	0.283	NR	1.416	0.139
6 months post-dose 2	0.601	0.200		0.207	0.241	0.240	0.179	0.109
Post-dose 3	5.886	1.706		2.075	2.081	1.008	2.965	0.370
6 months post-dose 3	--	0.762		0.733	0.169	0.195	1.423	0.516

[1] Data with fewer than five IDP at a given timepoint are not reported due to a lack of statistical confidence in the IDP-specific median.
[2] Data are not presented for the six months post-dose 3 timepoint as healthy volunteers were not eligible for a third dose during the study period, per vaccination guidelines from the Centers for Disease Control and Prevention.
[3] This column includes all IDP in the study.

Abbreviations: HVs, healthy volunteers; IDP, immune-deficient/disordered people; IgG; immunoglobulin G; NR, not reported

Table S6. Kendall correlation values of anti-S IgG antibody concentration and ACE2 pseudo-neutralization (% inhibition) by immunological subgroups. The correlations summarize data across six SARS-CoV-2 variants (*i.e.*, Ancestral, Alpha, Beta, Gamma, Delta, and Omicron BA.1) and four timepoints (*i.e.*, post-dose 2, six months post-dose 2, post-dose 3, and six months post-dose 3)

**Immunological subgroup**	**Correlation**	**95% BC_a CI[1]**
Healthy volunteers	0.57	(0.44, 0.73)
Antibody deficiency	0.55	(0.34, 0.69)
PIRD	0.47	(0.31, 0.63)
Combined immunodeficiency[2]	0.33	(0.02, 0.57)
Other IEI	0.69	(0.57, 0.80)
Other immune disorder	0.49	(0.31, 0.61)

[1]Data were bootstrapped at the individual level to account for intra-participant correlation.
[2]Due to few participants with combined immunodeficiencies in the study, these CIs are wider than for other immunological subgroups.

Abbreviations: ACE2, angiotensin converting enzyme 2; BC_a, bias-corrected and accelerated; CI, confidence interval; IEI, inborn errors of immunity; IgG, immunoglobulin G; PIRD, primary immune regulatory disorder; SARS-CoV-2, severe acute respiratory syndrome coronavirus 2

Table S7. Kendall correlation values of anti-S IgG antibody concentration and ACE2 pseudo-neutralization (% inhibition) by timepoints. The correlations summarize data across six immunological subgroups (*i.e.*, healthy volunteers, antibody deficiencies, PIRD, combined immunodeficiencies, other IEI, other immune disorders) and six SARS-CoV-2 variants (*i.e.*, Ancestral, Alpha, Beta, Gamma, Delta, and Omicron BA.1).

Timepoint	Correlation	95% BC_a CI[1]
Post-dose 2	0.57	(0.43, 0.68)
Six months post-dose 2	0.26	(0.09, 0.43)
Post-dose 3	0.67	(0.58, 0.74)
Six months post-dose 3	0.28	(0.06, 0.47)
One month after vaccination<br>(Combining post-dose 2 and post-dose 3)	0.66	(0.58, 0.72)
Six months after vaccination<br>(Combining six months post-dose 2 and six months post-dose 3)	0.32	(0.19, 0.44)

[1]Data were bootstrapped at the individual level to account for intra-participant correlation.

Abbreviations: ACE2, angiotensin converting enzyme 2; BC_a, bias-corrected and accelerated; CI, confidence interval; IEI, inborn errors of immunity; IgG, immunoglobulin G; PIRD, primary immune regulatory disorders; SARS-CoV-2, severe acute respiratory syndrome coronavirus 2

Table S8. Kendall correlation values of anti-S IgG antibody concentration and ACE2 pseudo-neutralization (% inhibition) by SARS-CoV-2 variants. The correlations summarize data across six immunological subgroups (*i.e.*, healthy volunteers, antibody deficiencies, PIRD, combined immunodeficiencies, other IEI, other immune disorders) and four timepoints (*i.e.*, post-dose 2, six months post-dose 2, post-dose 3, and six months post-dose 3)

SARS-CoV-2 variant	Correlation	95% BC_a CI[1]
Ancestral	0.60	(0.53, 0.67)
Alpha	0.60	(0.54, 0.66)
Beta	0.52	(0.44, 0.59)
Gamma	0.53	(0.45, 0.61)
Delta	0.58	(0.51, 0.65)
Omicron BA.1	0.25	(0.14, 0.34)

[1]Data were bootstrapped at the individual level to account for intra-participant correlation.

Abbreviations: ACE2, angiotensin converting enzyme 2; BC_a, bias-corrected and accelerated; CI, confidence interval; IEI, inborn errors of immunity; IgG, immunoglobulin G; PIRD, primary immune regulatory disorders; SARS-CoV-2, severe acute respiratory syndrome coronavirus 2

Table S9. Kendall correlation values of anti-S IgG antibody concentration and ACE2 pseudo-neutralization (% inhibition) by immunological subgroups and SARS-CoV-2 variants. The correlations summarize data across four timepoints (*i.e.*, post-dose 2, six months post-dose 2, post-dose 3, and six months post-dose 3).

Immunological subgroup	SARS-CoV-2 variant	Correlation	95% BC_a CI[1]
Healthy volunteers	Ancestral	0.68	(0.53, 0.87)
	Alpha	0.64	(0.48, 0.84)
	Beta	0.52	(0.32, 0.71)
	Gamma	0.61	(0.44, 0.82)
	Delta	0.55	(0.37, 0.74)
	Omicron BA.1	0.23	(0.01, 0.44)
Antibody deficiency	Ancestral	0.60	(0.30, 0.82)
	Alpha	0.63	(0.42, 0.81)
	Beta	0.56	(0.32, 0.74)
	Gamma	0.55	(0.29, 0.75)
	Delta	0.58	(0.29, 0.75)
	Omicron BA.1	0.33	0.02, 0.57)
Primary immune regulatory disorder	Ancestral	0.57	(0.40, 0.73)
	Alpha	0.54	(0.38, 0.72)
	Beta	0.45	(0.25, 0.62)
	Gamma	0.45	(0.24, 0.65)
	Delta	0.52	(0.33, 0.70)
	Omicron BA.1	0.11	(-0.20, 0.34)
Combined immunodeficiency[2]	Ancestral	0.32	(-0.43, 0.80)
	Alpha	0.45	(-0.32, 0.78)
	Beta	0.37	(-0.48, 0.76)
	Gamma	0.37	(-0.48, 0.76)
	Delta	0.37	(-0.48, 0.76)
	Omicron BA.1	-0.07	(-0.69, 0.50)
Other inborn error of immunity	Ancestral	0.75	(0.61, 0.93)
	Alpha	0.72	(0.55, 0.92)
	Beta	0.72	(0.58, 0.90)
	Gamma	0.77	(0.64, 0.92)
	Delta	0.7	(0.65, 0.94)
	Omicron BA.1	0.53	(0.23, 0.86)
Other immune disorder	Ancestral	0.55	(0.34, 0.69)
	Alpha	0.59	(0.41, 0.71)
	Beta	0.50	(0.29, 0.66)
	Gamma	0.49	(0.30, 0.64)
	Delta	0.56	(0.37, 0.71)

	Omicron BA.1	0.26	(0.03, 0.43)

[1]Data were bootstrapped at the individual level to account for intra-participant correlation.
[2]Due to the few participants with combined immunodeficiencies in the study, these data could not be bootstrapped. A bootstrap (at the sample level rather than at the individual level) 95% CI is therefore given. As a result of the few participants, these CIs are wider than for other immunological subgroups.

Abbreviations: ACE2, angiotensin converting enzyme 2; BC_a, bias-corrected and accelerated; CI, confidence interval; IgG, immunoglobulin G; SARS-CoV-2, severe acute respiratory syndrome coronavirus 2

Table S10. The causative SARS-CoV-2 variant of breakthrough infections.

Variant	Variant identified by sequencing (N=11)	Variant assigned based on national CDC estimates at the time of infection (N=24)
Delta	1	3
Omicron BA.1	7	17
Omicron BA.2	3	4

Abbreviations: CDC, Centers for Disease Control and Prevention; SARS-CoV-2, severe acute respiratory syndrome coronavirus 2

Table S11. Adverse events in IDP and HVs.

Dose	Adverse event information	HV	IDP
1	N	33	169
	Any local adverse event	29	126
	Any systemic adverse event	18	94
	ER visits	0	1
	Time from vaccination to symptom onset (median, days)	1	1
	Symptom duration (median, days)	2	2
2	N	30	165
	Any local adverse event	24	126
	Any systemic adverse event	22	108
	ER visits	0	0
	Time from vaccination to symptom onset (median, days)	1	1
	Symptom duration (median, days)	2	2
3	N	17	130
	Any local adverse event	12	95
	Any systemic adverse event	9	88
	ER visits	1	3
	Time from vaccination to symptom onset (median, days)	1	1
	Symptom duration (median, days)	1	2

Adverse events data comes from self-reported surveys. Not all participants completed every adverse event survey after each dose.

Abbreviations: ER, emergency room; HVs, healthy volunteers; IDP, immune-deficient/disordered people
